# Mendelian non-syndromic and syndromic hearing loss genes contribute to presbycusis

**DOI:** 10.1038/s41431-025-01789-x

**Published:** 2025-03-07

**Authors:** Diana M. Cornejo-Sanchez, Thashi Bharadwaj, Rui Dong, Gao T. Wang, Isabelle Schrauwen, Andrew T. DeWan, Suzanne M. Leal

**Affiliations:** 1 Center for Statistical Genetics, Gertrude H. Sergievsky Center, and the Department of Neurology, Columbia University Medical Center, New York, NY USA; 2 Department of Chronic Disease Epidemiology and Center for Perinatal, Pediatric and Environmental Epidemiology, Yale School of Public Health, New Haven, CT USA; 3 Taub Institute for Alzheimer’s Disease and the Aging Brain, Columbia University Medical Center, New York, NY USA; 4 Present Address: Department of Translational Neurosciences, University of Arizona College of Medicine Phoenix, Phoenix, AZ USA

**Keywords:** Genome-wide association studies, Genetics research, Data processing

## Abstract

Age-related (AR) hearing loss (HL) is the most prevalent sensorineural disorder in older adults. Here we demonstrate that rare-variants in well-established Mendelian HL genes play an important role in ARHL etiology. In all we identified 32 Mendelian HL genes which are associated with ARHL. We performed single and rare-variant aggregate association analyses using exome data obtained from white-Europeans with self-reported hearing phenotypes from the UK Biobank. Our analysis revealed previously unreported associations between ARHL and rare-variants in Mendelian non-syndromic and syndromic HL genes, including *MYO15A*, and *WFS1*. Additionally, rare-variant aggregate association analyses identified associations with Mendelian HL genes i.e., *ACTG1*, *GRHL2*, *KCNQ4*, *MYO7A*, *PLS1*, *TMPRSS3*, and *TNRC6B*. Four novel ARHL genes were also detected: *FBXO2* and *PALM3*, implicated in HL in mice, *TWF1*, associated with HL in Dalmatian dogs, and *TXNDC17*. In-silico analyses provided further evidence of inner ear expression of these genes in both murine and human models, supporting their relevance to ARHL. Analysis of variants with minor allele frequency >0.005 revealed additional ARHL associations with known e.g., *ILDR1* and novel i.e., *ABHD12, COA8*, *KANSL1*, *SERAC1*, and *UBE3B* Mendelian non-syndromic and syndromic HL genes as well as ARHL associations with genes that have not been previously reported to be involved in HL e.g., *VCL*. Rare-variants in Mendelian HL genes typically exhibited higher effect sizes for ARHL compared to those in other associated genes. In conclusion, this study highlights the critical role Mendelian non-syndromic and syndromic HL genes play in the etiology of ARHL.

## Introduction

Age-Related (AR) Hearing Loss (HL), commonly known as presbycusis, constitutes a widespread sensory deficit that affects >50% of individuals over 75 years-of-age. Men are twice as likely to be affected as females. ARHL can reduce quality of life and lead to social isolation making it a public health priority [1–3].

The etiology of ARHL is multifactorial, with genetic and environmental determinants [4]. Environmental risk factors for ARHL include noise exposure and ototoxic drugs [5]. Genome-wide association studies of ARHL that have predominantly targeted common variants have found associations with ~80 genes [5–8], although many have not been replicated.

This study focuses on the contribution of rare-variants to the genetic architecture of ARHL, using self-reported HL phenotypes and exome-sequence data obtained from ~460,000 white-European UK Biobank participants, which provided additional power to detect associations compared to our previous study that only included the exome-sequence data that was available at that time (*N* = ~170,000) [9].

Our analysis found ARHL rare-variant associations with Mendelian non-syndromic (NS) and syndromic (S) HL genes. We also observed, rare-variant associations with genes that have not been previously reported to be involved in human HL but have been implicated in HL in mice and Dalmatian dogs. For these genes, *in-silico* evidence of inner ear expression, notably within hair cells, in both murine and human models further corroborate their relevance to HL. We also identified association with variants with a minor allele frequency (MAF) > 0.005 in several Mendelian NSHL and SHL genes. This study offers new insights into the genetic etiology of ARHL, highlighting the important role of Mendelian HL genes.

## Subjects and methods

### Data access and ethical approval

This research was conducted using UK Biobank data (application number 32285) and their Research Analysis Platform. The UK Biobank study was conducted under generic approval from the National Health Services’ National Research Ethics Service. The present analyzes were approved by the Institutional Review Boards at Yale University (2000026836) and Columbia University (AAAS3494).

### Quality control of genotype-array data and samples

For the genotype-array data (field 22418), variants not present in both the UK BiLEVE and UK Biobank Axiom arrays, or that failed the UK Biobank batch quality control, non-autosomal, missing genotype data i.e., >5% (variants with MAF > 5%) and >1% (variants with MAF ≤ 5%), or failed Hardy-Weinberg equilibrium ($p \, < 1.0\,\times {10}^{-15}$) were removed. Individuals with inconsistent sex (self-reported vs genomic) or sex aneuploidies and poor-quality samples (field 22027) were also removed.

Using the genotype-array data, principal component (PC) analysis was performed using FlashPCA2 to determine which individuals are of white-European ancestry. Ten PCs were constructed using unrelated individuals (Θ_ij_ < 0.0625 - estimated using KING), related individuals were projected back onto the PCs. Mahalanobis distances were calculated and 0.3% of the samples with the most extreme values were removed.

### Quality control for exome-sequence data

The exome-sequence data (field 23157) were generated using the functional equivalence pipeline (OQFE protocol) [10]. Bcftools v.1.16 [11] was used to perform multiallelic splitting, alignment, and indel left-normalization. Genotypes with genotype quality<20 and/or read depth <8 for SNVs and <10 for indels were removed. A variant-level allele-balance filter was applied, retaining variants that had either ≥1 homozygous or ≥1 heterozygous genotype with an allele-balance ≥0.15 for SNVs or ≥0.20 for indels. Variant sites missing >10% of their data were also removed.

### Determining case/control status

ARHL phenotypes were defined as previously described [9]. In brief, ICD10/9 and self-report codes were used to avoid the inclusion of individuals with environmental forms of HL, early-onset (<55 years-of-age or no age-of diagnosis), or late-onset HL, which is not presbycusis, e.g. Meniere’s, otosclerosis.

Individuals who did not have any ICD10/9 codes for HL and consistently reported to not have: (i) ***H-aid*** use of hearing aids (f.3393), (ii) ***H-diff*** hearing difficulty (f.2247), or (iii) ***H-noise*** hearing difficulty with background noise (f.2257) formed the common set of controls (*N* = 231,960). Completely deaf individuals were not asked f.3393, f.2247, and f.2257. Cases were individuals with ***H-aid***, ***H-diff***, ***H-noise***, or ***H-both*** (reported having H-diff and H-noise) that did not meet the exclusion criteria and provided consistent answers. The number of cases is *N* = 16,854 (***H-aid)***, *N* = 110,249 (***H-diff)***, *N* = 160,150 (***H-noise)***, and *N* = 93,410 (***H-both)****.*

Controls age at last visit and cases age when they first reported ***H-aid***, ***H-diff***, or ***H-noise*** were included as a covariate in the analysis*.* The first two PCs (obtained from genotype-array data) for each ARHL phenotype were recalculated, e.g., **H-aid** cases and control and used as covariates in the analysis.

### Variant annotation

ANNOVAR and VEP were used to annotate variants obtained from the exome-sequence data (GRCh38). Gene regions, variant function, amino acid change, Combined Annotation Dependent Depletion (CADD) Phred scores, and gnomAD non-Finnish European (NFE) allele frequencies were annotated. Variants were classified as predicted loss-of-function (pLoF) if they were annotated as frameshift, stop-loss, stop-gain, start-loss, or were located within ±2 base-pairs (bp) of the splice-site. Splice-site variants which were not annotated as pLoF were those variants >±2 bp but not more than ±12 bp from the splice-site (from here on defined as splice-region variants).

### Association analysis

Single and rare-variant aggregate association testing for each ARHL phenotype was conducted using a generalized linear mixed model approximation as implemented in REGENIE v.3.4 [12]. In the first step, the ridge regression model was fitted using genotype-array variants. In the second step, association testing was performed using the Firth correction. For the single-variant analysis, those with a minor allele count ≥4, were analyzed. A genome-wide significant level of $p \, < \,{5.0\times 10}^{-8}$ was used. Rare-variant aggregate tests were used to analyze genes with ≥2 rare variants (NFE MAF≤0.005 in gnomAD v3.12 and v2.1, these versions of gnomAD exclude UK Biobank study subjects) using both a burden test and the optimal sequence kernel association test (SKAT-O) [13]. The variants which were included in the aggregate tests were either 1.) pLoF or 2.) pLoF and missense and splice-region with a CADD score ≥20. For the burden test and the burden component of SKAT-O, a 0, 1, 2 coding was used for no rare-variants, ≥1 heterozygous rare-variant, or ≥1 homozygous rare-variant within a region, respectively. An exome-wide significance level of $p < 2.5\times {10}^{-6}$ was used (a Bonferroni correction for testing 20,000 genes). Age, sex, and two PCs were included as covariates in both the single and rare-variant aggregate association models.

### In-silico expression of novel genes associated with ARHL in human and mouse inner ear

RNA sequence (RNAseq) expression levels of *FBXO2*, *PALM3*, *TWF1*, and *TXNDC17* in the mouse and human inner ear were obtained and visualized using publicly available datasets from gEAR (gene Expression Analysis Resource) [14], i.e., single-nucleus RNAseq data from two human fetal (7.5 and 9.2 weeks) and one adult inner ears (accession number GSE213796), single-cell (sc) and single-nucleus RNAseq data obtained from human inner ear organoids (accession number GSE214099) [15], and scRNAseq data obtained from CD-1 mice cochleae at development stages E14, E16, P1, and P7 (accession number GSE137299) [15, 16].

## Results

### Rare single-variant association analysis

Single-variant analysis revealed significant associations for variants in Mendelian genes across all the ARHL phenotypes (Table 1, Figures S1 and S2). For instance, the pathogenic variant rs121912560 in the Mendelian HL gene *MYO6* was associated with all ARHL traits $[p \, < \,1.5\times {10}^{-12};{{\rm{odds}}}\; {{\rm{ratio}}}({{\rm{OR}}})=17.1-166.0].$ Similarly, rs1459651793 in *COL11A2*, a gene implicated in autosomal dominant NSHL (ADNSHL) and autosomal recessive NSHL (ARNSHL), was significantly associated with all ARHL traits $(p \, < \,1.0\times {10}^{-13};{{\rm{OR}}}=6.6{{-}}28.1).$ Additionally, rs761934676 in *TBC1D24*, a gene involved in ADNSHL, showed significant associations ($p \, < \,5.0\times {10}^{-9}$) with all the traits except for H-noise, with H-aid having the highest effect estimate (OR = 20.4). Variants rs370598077 and rs141952919 in *SCL26A5*, a gene linked to ARNSHL, were associated with H-diff and H-both, with rs370598077 also associated with H-noise, all with ORs ranging between 1.2 and 1.3. Rs367547063 in *WFS1*, a gene implicated in Wolfram and Wolfram-like syndromes and ADNSHL, was significantly associated with H-aid (OR = 10.0) and H-both (OR = 4.1). Rs2126961780 in *POU4F3*, known to contribute to ADNSHL, was associated with H-aid ($p \, < \,3.5\times {10}^{-10};{{\rm{OR}}}=603.3$). Variants rs186687142 and rs191552868 in *CEACAM16*, which is involved in ADNSHL and ARNSHL, were also associated with H-aid ($p \, < \, 5.0\times {10}^{-8};{{\rm{OR}}}=2.1\,{{\rm{for}}} \, {{\rm{both}}} \, {{\rm{variants}}}$). Rs876657898 (a variant of unknown significance for ARNSHL) in *MYO15A*, was associated with H-both ($p \, < \,5.0\times {10}^{-8};{{\rm{OR}}}=1.6$).

**Table 1:** Genome-wide significant rare-variant (MAF ≤ 0.005) associations with age-related hearing loss.

A. Variants associated with ARHL in Mendelian HL genes																
Chr	Gene	Variant	rsID	EAF	H-aid			H-diff			H-noise			H-both		HL Phenotypes ^a,b^
					*β* (SE)	*P*		*β* (SE)		*P*	*β* (SE)	*P*		β(SE)	*P*	
4	*WFS1*	c.2470G>A:p.(E824K)	rs367547063	9.4 × 10^−5^	2.30(0.38)		2.0 × 10^−8^*	1.29(0.26)	4.3 × 10^−7^		0.91(0.25)		1.7 × 10^−4^	1.41(0.26)	3.4 × 10^−8^*	Wolfram Syndrome 1, DFNA6/14/38
5	*POU4F3*	c.694G>C:p.(E232Q)	rs2126961780	1.1 × 10^−5^	6.40(1.65)		3.5 × 10^−10^**	3.57(1.58)	1.6 × 10^−4^		3.17(1.55)		7.7 × 10^−4^	3.69(1.58)	7.9 × 10^−5^	DFNA15
6	*COL11A2*	c.239T>C:p.(F80S)	rs1459651793	1.0×10^-4^	3.34(0.40)		2.2 × 10^−16^**	2.25(0.30)	1.2 × 10^−18^**		1.88(0.30)		1.0 × 10^−13^**	2.31(0.30)	1.2 × 10^−18^**	DFNA13, DFNB53, ARHL^c^
6	*MYO6*	c.737A>G:p.(H246R)	rs121912560	5.4 × 10^−5^	5.11(0.66)		5.3 × 10^−20^**	3.28(0.57)	6.7 × 10^−17^**		2.84(0.57)		1.4 × 10^−12^**	3.31(0.57)	2.1 × 10^−16^**	DFNA22, DFNB37, ARHL^c,d^
7	*SLC26A5*	g.103411631T>C	rs370598077	3.8 × 10^−3^	0.29(0.09)		1.9 × 10^−3^	0.25(0.04)	8.2 × 10^−9^**		0.21(0.04)		4.2 × 10^−8^*	0.28(0.04)	3.0 × 10^−10^**	DFNB61
7	*SLC26A5*	c.137T>C:p.(L46P)	rs141952919	5.3 × 10^−3^	0.30(0.08)		1.2 × 10^−4^	0.22(0.04)	8.0 × 10^−10^**		0.17(0.03)		1.2 × 10^−7^	0.25(0.04)	2.2 × 10^−11^**	DFNB61
16	*TBC1D24*	c.920A>G:p.(N307S)	rs761934676	9.5 × 10^−5^	3.02(0.36)		4.7 × 10^−14^**	1.46(0.26)	4.8 × 10^−9^**		1.07(0.25)		1.1 × 10^−5^	1.57(0.26)	5.7 × 10^−10^**	DFNA65, ARHL^c^
17	*MYO15A*	c.346_355delinsG: p.(Y117_R119del)	rs876657898	1.2 × 10^−3^	0.83(0.14)		5.7 × 10^−8^	0.40(0.08)	1.9 × 10^−7^		0.36(0.07)		2.1 × 10^−7^	0.45(0.08)	1.7 × 10^−8^*	DFNB3
19	*CEACAM16*	c.95G>T:p.(S32I)	rs186687142	1.5 × 10^−3^	0.75(0.13)		3.1 × 10^−8^*	0.32(0.07)	3.4 × 10^−6^		0.24(0.06)		8.2 × 10^−5^	0.34(0.07)	2.1 × 10^−6^	DFNA4B, DFNB113
19	*CEACAM16*	c.96C>T:p.(S32S)	rs191552868	1.5 × 10^−3^	0.76(0.13)		2.7 × 10^−8^*	0.32(0.07)	2.5 × 10^−6^		0.25(0.06)		5.1 × 10^−5^	0.35(0.07)	1.6 × 10^−6^	DFNA4B, DFNB113

B. Variants associated with ARHL in novel genes																
5	*PPWD1*	g.65572309C > T	rs113550012	4.0 × 10^-5^	4.00(0.66)		3.5 × 10^−8^*	1.68(0.48)	1.9 × 10^−4^		1.59(0.43)		4.4 × 10^−5^	1.85(0.48)	4.1 × 10^−5^	
16	*ZNF598*	c.443G>A:p.(R148H)	rs746426951	8.6 × 10^−5^	3.13(0.38)		6.7 × 10^−14^**	1.45(0.27)	4.2 × 10^−8^*		1.11(0.27)		1.5 × 10^−5^	1.54(0.28)	1.8 × 10^−8^*	

C. Variants in genes which have previously been associated with ARHL but not known to cause Mendelian HL																
5	*PDCD6*	c.132A>G:p.(S44S)	rs537688122	5.4 × 10^−4^	1.85(0.19)		6.5 × 10^−19^**	1.32(0.11)	8.7 × 10^−34^**		1.06(0.11)		5.7 × 10^−25^**	1.37(0.11)	5.8 × 10^−34^**	
5	*PDCD6*	c.139G>C:p.(E47Q)	rs549592074	1.9 × 10^−4^	1.89(0.30)		4.8 × 10^−9^**	1.36(0.18)	1.9 × 10^−14^**		0.98(0.18)		1.6 × 10^−8^*	1.38(0.19)	7.1 × 10^−14^**	
5	*PDCD6*	c.146A>G:p.(Q49R)	rs571370281	4.2 × 10^−4^	1.73(0.22)		3.4 × 10^−13^**	1.34(0.12)	3.2 × 10^−29^**		1.02(0.12)		1.6 × 10^−18^**	1.38(0.13)	1.3 × 10^−28^**	
6	*FILIP1*	g.75362956T>C	rs765264064	9.9 × 10^−5^	2.51(0.37)		1.1 × 10^−9^**	1.38(0.26)	3.1 × 10^−8^*		1.11(0.25)		3.1 × 10^−6^	1.38(0.26)	1.3 × 10^−7^	
22	*NCAPH2*	g.50517688G>A	rs200126237	1.1 × 10^−3^	0.81(0.15)		4.3 × 10^−7^	0.45(0.08)	1.8 × 10^−8^*		0.35(0.07)		2.0 × 10^−6^	0.50(0.08)	2.7 × 10^−9^**	
22	*KLHDC7B*	p.G943Afs*34	rs749405486	5.8 × 10^−4^	1.42(0.18)		8.1 × 10^−13^**	0.83(0.10)	2.7 × 10^−15^**		0.64(0.10)		3.5 × 10^−11^**	0.90(0.11)	1.3 × 10^−16^**	

*ARHL* age-related hearing loss, *Chr* Chromosome, *EAF* effect allele frequency for H-both sample cases and controls (*N* =  392,110), the allele frequencies for the other phenotypes were very similar with a slight change due to differences in sample size, *SE* standard error, *P* *P*-value.

*Variants that are genome-wide significant ($p < 5.0\times {10}^{-8}$) or **are genome-wide significant after adjusting for testing four phenotypes ($p < 1.25\times {10}^{-8}$);, i.e., hearing aid (H-aid), hearing difficulty (H-diff), hearing difficulty with background noise (H-noise) or the combined hearing trait (H-both) in the univariate analysis of the white-European individuals from the UK Biobank.

^a^DFNA: Autosomal dominant non-syndromic hearing loss locus.

^b^DFNB: Autosomal recessive non-syndromic hearing loss locus.

^c^Praveen et al. (PMID: 35661827).

^d^Cornejo-Sanchez et al. (PMID 36888145).

Previously reported associations for ARHL with *PDCD6*, *FILIP1*, *NCAPH2*, and *KLHDC7B*, were also observed.

Finally, novel significant associations were observed for rs113550012 in *PPWD1* with H-aid (OR = 54.4) and for rs746426951 in *ZNF598* with H-aid (OR = 22.8), H-diff (OR = 4.3) and H-both (OR = 4.6) which are both located near Mendelian HL genes. The associated variant in *ZNF598* is in LD (*r*^2^ = 0.67) with rs761934676 in *TBC1D24*, while the *PPWD1* variant is not in LD with any variants in *POU4F3*.

### Rare-variant aggregate association analysis

**Fig. 1:**
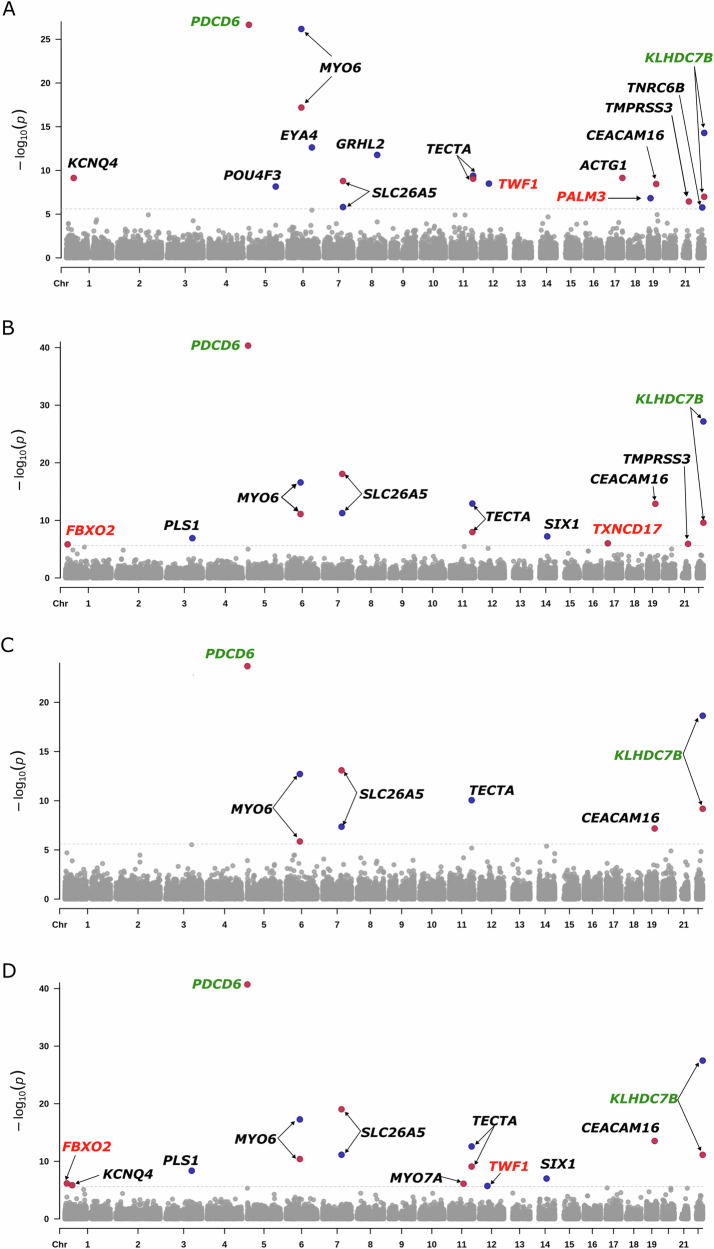
Manhattan plots for the rare-variant aggregate association analysis using SKAT-O. Results are shown for the analysis of H-aid (**A**), H-diff (**B**), H-noise (**C**), and H-both (**D**). In each Manhattan plot, blue dots represent the results for the analysis of predicted loss of function (pLoF) variants, while the red dots indicate the results for the combined analysis of pLoF, missense (${\mbox{CADD}}\ge 20$) and splice-region variants (${\mbox{CADD}}\ge 20$). The threshold for exome-wide significance ($p < 2.5\times {10}^{-6}$) is marked by a dashed gray line. Genes that achieved exome-wide significance in either analysis are annotated. Known Mendelian hearing loss genes are highlighted in black, known age-related hearing loss (ARHL) genes in green, and novel ARHL genes in red.

Rare-variant aggregate association analysis of ARHL revealed associations with known Mendelian HL genes. All significant results were obtained from the analysis performed using SKAT-O (Table 2 and Fig. 1) and some of these results were no longer significant when a burden test was performed (Table S1 and Figure S4). Many of these associations were only observed when pLoF variants were analyzed. *MYO6* was significantly associated (OR = 2.3–11.5) with all ARHL traits in both the burden and SKAT-O analyses. Similarly, *SLC26A5* was significantly associated (OR = 2.7–6.0), with all four traits for both tests. *TECTA*, an ADNSHL and ARNSHL gene, was also (OR = 1.7–3.1) significantly associated across all ARHL traits and analyzes. For each of these genes the highest OR was observed for H-aid. *PLS1* and *SIX1*, genes known for their roles in ADNSHL, were significantly associated with H-diff and H-both. *PLS1* was significant in the burden (OR = 2.0) and SKAT-O analyses, while *SIX1* was associated only in the SKAT-O analysis. *EYA4* (OR = 10.6), *GRHL2* (OR = 50.2), and *POU4F3* (OR = 488.1) were associated with H-aid in both the burden and SKAT-O analysis. SKAT-O revealed an association with H-aid for *TNRC6B*, a gene implicated in developmental delay which can also present with HL.

**Table 2:** SKAT-O rare-variant aggregated association results with age-related hearing loss phenotypes.

pLoF variants						
Mendelian HL genes associated with ARHL						
Chr	Gene	H-aid *p*-value	H-diff *p*-value	H-noise *p*-value	H-both *p*-value	HL Phenotypes^a,b^
3	*PLS1*	5.71 × 10^−^^5^	1.22 × 10^−7**^	2.96 × 10^−6^	4.32 × 10^−9**^	DFNA76
5	*POU4F3*	6.97 × 10^−9**^	1.43 × 10^−4^	3.92 × 10^−4^	5.30 × 10^−5^	DFNA15, ARHL^c^
6	*MYO6*	6.74 × 10^−27**^	2.60 × 10^−17**^	1.93 × 10^−13**^	5.30 × 10^−18**^	DFNA22, DFNB37, ARHL^c,d^
6	*EYA4*	2.37 × 10^−13**^	3.59 × 10^−4^	1.87 × 10^−2^	2.94 × 10^−4^	DFNA10, ARHL^c,d^
7	*SLC26A5*	1.55 × 10^−6*^	5.41 × 10^−12**^	4.35 × 10^−8**^	7.23 × 10^−12**^	DFNB61, ARHL^d^
8	*GRHL2*	1.69 × 10^−12**^	1.58 × 10^−4^	7.25 × 10^−3^	7.26 × 10^−5^	DFNA28
11	*TECTA*	4.03 × 10^−10**^	1.22 × 10^−13**^	8.80 × 10^−11**^	2.63 × 10^−13**^	DFNA8/12, DFNB21, ARHL^c,d^
14	*SIX1*	2.04 × 10^−5^	6.03 × 10^−8**^	4.10 × 10^−6^	9.81 × 10^−8**^	DFNA23, BORS^e^, ARHL^c^
22	*TNRC6B*	1.71 × 10^−6*^	7.52 × 10^−3^	2.77 × 10^−2^	1.67 × 10^−3^	Developmental Delay^f^
**Novel genes associated with ARHL**						
12	*TWF1*	3.20 × 10^−9**^	7.3 × 10^−6^	1.60 × 10^−4^	1.90 × 10^−6*^	HL in Dalmatian dogs^g^
19	*PALM3*	1.49 × 10^−7**^	1.78 × 10^−4^	1.69 × 10^−3^	1.96 × 10^−5^	Mouse HL gene^h^
**Genes previously associated with ARHL that are not know to be involved in Mendelian HL**						
22	*KLHDC7B*^*3,4*^	5.11 × 10^−15**^	6.65 × 10^−28**^	2.29 × 10^−19**^	3.23 × 10^−28**^	

pLoF, missense and splice-region variants with CADD ≥ 20						
**Mendelian HL genes associated with ARHL**						
1	*KCNQ4*	7.28 × 10^−10***^	1.40 × 10^−5^	1.30 × 10^−4^	1.41 × 10^−6*^	DFNA2A
6	*MYO6*	6.40 × 10^−18***^	7.87 × 10^−12***^	1.38 × 10^−6*^	3.99 × 10^−11***^	DFNA22; DFNB37, ARHL^c,d^
7	*SLC26A5*	1.61 × 10^−9***^	8.60 × 10^−19***^	8.06 × 10^−14***^	9.09 × 10^−20***^	DFNB61, ARHL^d^
11	*MYO7A*	1.24 × 10^−5^	3.46 × 10^−6^	6.36 × 10^−5^	7.82 × 10^−7**^	DFNA11; DFNB2; Usher syndrome
11	*TECTA*	8.88 × 10^−10***^	1.07 × 10^−8***^	6.55 × 10^−6^	8.12 × 10^−10***^	DFNA8/12; DFNB21, ARHL^c,d^
17	*ACTG1*	7.17 × 10^−10***^	8.50 × 10^−5^	6.91 × 10^−3^	2.67 × 10^−5^	DFNA20/26
19	*CEACAM16*	3.58 × 10^−9***^	1.31 × 10^−13***^	6.62 × 10^−8***^	2.94 × 10^−14***^	DFNA4B; DFNB113, ARHL^c^
21	*TMPRSS3*	3.52 × 10^−7***^	1.19 × 10^−6**^	3.42 × 10^−4^	4.63 × 10^−6^	DFNB8/10
**Novel genes associated with ARHL**						
1	*FBXO2*	1.20 × 10^−4^	1.46 × 10^−6*^	1.98 × 10^−5^	6.79 × 10^−7**^	ARHL in mice^i^
17	*TXNDC17*	1.76 × 10^−2^	9.53 × 10^−7**^	2.34 × 10^−4^	4.14 × 10^−6^	
**Genes previously associated with ARHL that are not known to be involved in Mendelian HL**						
5	*PDCD6*^*4*^	2.21 × 10^−27***^	4.51 × 10^−41***^	2.09 × 10^−24***^	1.95 × 10^−41***^	
22	*KLHDC7B*^*3,4*^	1.03 × 10^−7***^	2.49 × 10^−10***^	6.58 × 10^−10***^	7.47 × 10^−12***^	

*Chr* chromosome, *p*-value for SKAT-O test, *H-aid* hearing aid, *H-diff* hearing difficulty, *H-noise* hearing difficulty with background noise, *H-both* hearing difficulty and hearing difficulty with background noise.

*Significant after Bonferroni correction for testing 20,000 genes ($p < 2.5\times {10}^{-6}$), **Significant after Bonferroni correction for testing 20,000 genes and testing two categories of variants, e.g., ($p < 1.25\times {10}^{-6}$), ***Significant after Bonferroni correction for testing 20,000 genes, four traits, and two categories of variants, e.g., pLoF ($p < 3.1\times {10}^{-7}$).

^a^DFNA: Autosomal dominant non-syndromic hearing loss locus.

^b^DFNB: Autosomal recessive non-syndromic hearing loss locus.

^c^Praveen et al. (PMID: 35661827).

^d^Cornejo-Sanchez et al. (PMID: 36788145).

^e^BORS: Branchio-oto-renal syndrome.

^f^with speech, behavioral abnormalities, and hearing loss.

^g^Candidate gene for congenital sensorineural deafness in Dalmatian dogs (PMID: 24324618).

^h^Personal communication Vogl and Kilimann as hearing loss gene in mice.

^i^Deletion of FBXO2 in mice leads to age-related hearing loss beginning by two months of age (PMID: 17494702).

Expanding the analysis to encompass pLoF combined with missense and splice-region variants with a CADD ≥ 20, revealed additional significant associations while others no longer remained significant. *MYO6* was no longer associated with H-noise for the burden test. Although the associations remained statistically significant for the other ARHL phenotypes, the *p*-values increased and the ORs decreased. *TECTA* was no longer significant for H-noise, and for the other traits the *p*-values were less significant, and the ORs decreased by ~50% compared to when only pLoF variants were analyzed. *SCL26A5* maintained its significance across all ARHL phenotypes and analyzes. However, there was a substantial decrease in the ORs, compared to those observed when only pLoF variants were analyzed. Even though the ORs decreased the *p*-values were more significant compared to when only pLoF variants were analyzed due to a smaller standard error. *CEACAM16*, which underlies ADNSHL and ARNSHL, although not significant when only pLoF variants were analyzed, displayed significant associations with all ARHL phenotypes (OR = 1.2–1.6) for burden and SKAT-O when pLoF and those missense and splice-region variants with a CADD ≥ 20 were included in the analysis.

*KCNQ4*, known for its role in ADNSHL, was associated with H-aid and H-both in the SKAT-O analysis. *MYO7A*, involved in ADNSHL, ARNSHL, and Usher syndrome type 1B, was associated with H-both only for SKAT-O. *TMPRSS3*, implicated in ARNSHL, was significantly associated with H-aid and H-diff in the SKAT-O but not the burden analysis. *ACTG1*, an ADNSHL gene, was associated with H-aid in both burden (OR = 5.1) and SKAT-O analyses.

In addition, associations were observed with genes that have not been implicated in HL in humans. Notably, pLoF variants in the *TWF1* gene, which is known to be associated with HL in Dalmatian dogs, were significantly associated with H-aid (OR = 13.7) and H-both (OR = 4.0) in both the burden and SKAT-O analyses. *PALM3*, which causes HL in knockout mice (personal communication Vogl and Kilimann 2024), showed a significant association only with H-aid in both burden (OR = 11.1) and SKAT-O analyses. After incorporating missense and splice-region variants (CADD ≥ 20) into the analysis, *FBXO2* and *TXNDC17*, reached exome-wide significance. *FBXO2*, a gene associated with ARHL in mice, was significantly associated with H-diff (OR = 1.4) and H-both (OR = 1.4) in both burden and SKAT-O analyses. *TXNDC17* was only associated with H-diff in the SKAT-O analysis*.* Significant associations were also observed with previously reported ARHL genes *KLHDC7B* and *PDCD6* [9].

### Single variant (MAF > 0.005) associations

ARHL associations were observed with variants in known NSHL genes, i.e., *CDH23*, *CLRN2*, *EYA4*, *GJB2*, *ILDR1*, and *TRIOBP* as well as with several SHL genes, i.e. *ABHD12*, *COA8*, *KANSL1*, *MLXIPL*, *SERAC1*, *SPTBN1, TYR*, and *UBE3B*. Of these Mendelian genes, *ABHD12, COA8*, *KANSL1, SERAC1*, and *UBE3B* have not been previously reported to be associated with ARHL. Additionally, variants in several genes not reported to be involved in Mendelian HL and/or presbycusis were found associated with ARHL, i.e., *ARHGAP27, CRHR1, GOLGB1, GRM3, HCLS1, LRRC37A2, LRRC37A3, MAPT, MMAB, NASP, NSF, PHF23, PLEKHM1, RTRAF, SPPL2C, STH, SYBU*, and *VCL*.

Finally, variants in genes previously implicated in ARHL were also found to be associated, i.e., *ABCC10, ACADVL, ARHGEF28*, *BAIAP2L2, CCDC17*, *CCDC68*, *CHMP4C*, *CRIP3*, *DVL2*, *GCAT*, *KLHDC7B*, *MAST2*, *PIK3R3, SLC22A7*, *SNAP91*, and *SYNJ2* (Table 3 and S2).

**Table 3:**
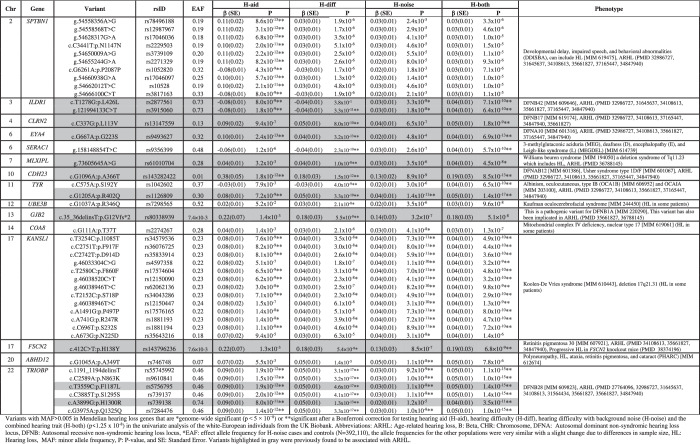
Variants (MAF > 0.005) in Mendelian Traits genes with genome-wide significant associations with age-related hearing loss phenotype.

Variants highlighted in gray were previously found to be associated with ARHL.

*ARHL* age-related hearing loss, *B* Beta, CHR chromosome, *DFNA* Autosomal dominant non-syndromic hearing loss locus, *DFNB* Autosomal recessive non-syndromic hearing loss locus, *EAF effect allele frequency for H-noise cases and controls (*N* = 392,110), the allele frequencies for the other populations were very similar with a slight change due to differences in sample size, *HL* Hearing loss, *MAF* minor allele frequency, *P* *P*-value, *SE* standard error.

Variants with MAF > 0.005 in Mendelian hearing loss genes that are *genome-wide significant (*p*  <  5 × 10^−8^) or **significant after a Bonferroni correction for testing hearing aid (H-aid), hearing difficulty (H-diff), hearing difficulty with background noise (H-noise) and the combined hearing trait (H-both) (*p* < 1.25 × 10^−8^) in the univariate analysis of the white-European individuals from the UK Biobank.

### In-silico mRNA expression of novel ARHL genes

**Fig. 2:**
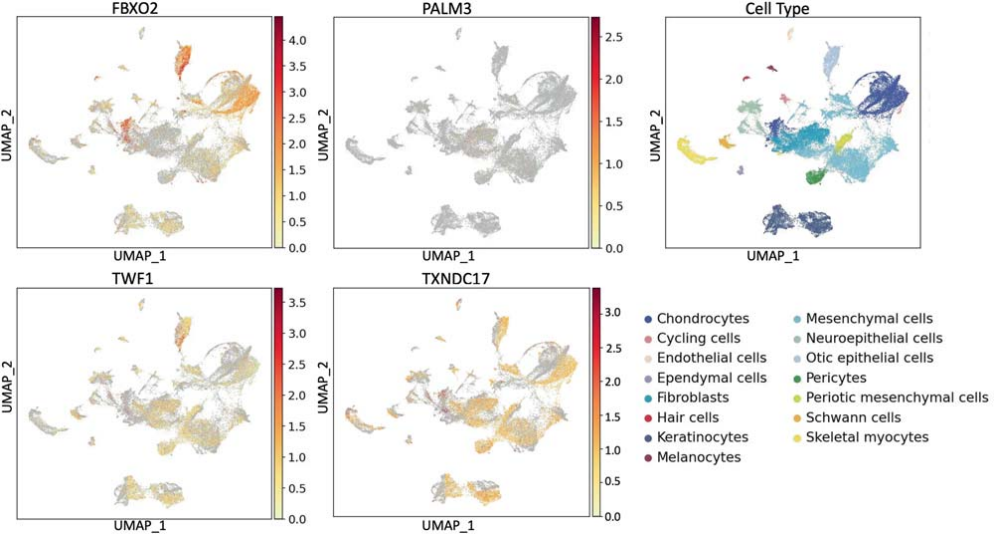
Expression of *FBXO2, PALM3*, *TWF1*, and *TXNDC17* in the human inner ear organoids. The dataset consists of human pluripotent stem cells developed and differentiated into complex inner ear tissue for which single-cell and single-nucleus RNA sequence data have been generated. The X-axis displays the uniform manifold approximation and projection (UMAP) 1 which is used for dimension reduction and on the Y-axis UMAP 2. The scale bar shown for each gene displays normalized gene expression levels which range from low (yellow) to high (red) that have been adjusted for sequence depth. The far-right panel displays the locations of specific inner ear cell types.

**Fig. 3:**
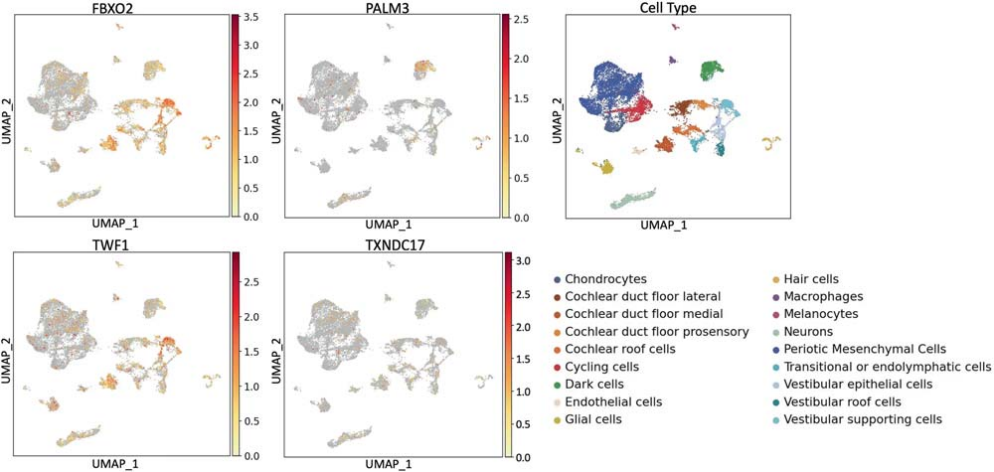
Expression of *FBXO2, PALM3*, *TWF1*, and *TXNDC17* in the human inner ear atlas. The dataset contains single-nucleus RNA sequence data from two human fetal inner ears (age 7.5 and 9.2 weeks) and one human adult inner ear. The X-axis displays the uniform manifold approximation and projection (UMAP) 1 which is used for dimension reduction and on the Y-axis UMAP 2. The scale bar shown for each gene displays normalized gene expression levels which range from low (yellow) to high (red) that have been adjusted for sequence depth. The far-right panel displays the locations of specific inner ear cell types.

We investigated the inner ear expression of *FBXO2*, *PALM3*, *TWF1*, and *TXNDC17*, genes which showed significant rare-variant aggregate associations with ARHL but have not been previously implicated in human HL. *FBXO2* exhibits low to moderate expression in most cells across the human inner ear with notable exceptions: high expression is found in chondrocytes and otic epithelial cells in human inner ear organoids (Fig. 2). Additionally, expression was observed in the human inner ear in the vestibular supporting and roof cells, hair cells, and cochlear duct floor cells (Fig. 3). In mice, the cochlear epithelium shows very high and widespread expression at E14, E16, P1, and P7 (Figures S6-S9).

In human inner ear organoids, *PALM3* expression is observed in fibroblasts and mesenchymal cells (Fig. 2). In the human inner ear, expression is observed in dark cells (responsible for inner ear fluid homeostasis), and vestibular and cochlear hair cells, neurons, cycling cells, and chondrocytes (Fig. 3). In the mouse inner ear, orthologue *Palm3* is highly localized to inner and outer hair cells. Expression levels of *Palm3* increase from E14 to E16, with notably higher levels during the post-natal stages P1 and P7 (Figures S6–S9).

In human inner ear organoid, *TWF1* is highly expressed specifically in otic epithelial cells, while a low expression is observed in most other cell types (Fig. 2). In the human inner ear, *TWF1* expression is observed in cycling cells, chondrocytes, dark cells, periotic mesenchymal cells, vestibular supporting cells, glial cells, neurons, and cochlear duct and roof cells (Fig. 3). In the mouse inner ear, *Twf1* expression in the cochlear epithelium is more pronounced compared to the human inner ear, with widespread expression that is slightly higher during stages P1 and P7 compared to E14 and E16 (Figures S6–S9).

The expression of *TXNDC17* is generally low to moderate in the human inner ear, though higher levels are observed in chondrocytes, keratinocytes, and fibroblasts within the inner ear organoids (Fig. 2). Expression is also present in chondrocytes and mesenchymal cells in the human inner ear (Fig. 3). In mice, the cochlear epithelium shows widespread and pronounced expression of *Txndc17* at E14, E16, P1, and P7 stages (Figures S6–S9).

## Discussion

We leveraged exome-sequence data from white-Europeans in the UK Biobank to conduct rare-variant aggregate and single-variant association analyses for ARHL and found that many rare-variants that increase ARHL susceptibility are within well-established Mendelian HL genes (e.g. *MYO6, PLS1*, *TECTA*). In total, we found associations between ARHL and rare-variants in 18 Mendelian HL genes. For variants with a MAF > 0.005 we found 14 additional genes which are involved the etiology of Mendelian NSHL and SHL. Rare pLoF variant aggregate association analysis also generally had higher ORs, than those aggregate association tests that also included missense and splice-region variants (CADD ≥ 20).

Many of the Mendelian HL genes we identified to be associated with ARHL are known to cause ADNSHL, and in some cases also ARNSHL and/or SHL. However, a few of these genes, i.e. *MYO15A, SLC26A5*, and *TMPRSS3*, are exclusively associated with ARNSHL. Some of these Mendelian HL genes i.e., *CEACAM16*, *COL11A2*, *EYA4*, *MYO6, POU4F3, SIX1, SLC26A5*, and *TECTA*, have previously been shown to be implicated in ARHL by both our research and others [7, 9]. There have also been reports of ARHL in single cases or families being due to variants in *COL11A2* and *WSF1* [17, 18].

Individuals with *POU4F3* variants can present with sensorineural, post-lingual, late onset, and progressive HL; additionally phenotypes are variable within families [19]. In our study, the variant rs2126961780 [c.694G>C:p.(E232Q)] in *POU4F3* was present in five cases with H-aid but was absent in all controls. Although this variant has not been classified as pathogenic in ClinVar, another variant c.694G>T:p.[(E232*)] that leads to an early stop-codon at the same genomic position, has been reported as pathogenic for ADNSHL [20], suggesting that different alleles within the same gene can cause either early-onset HL or ARHL.

*CEACAM16, MYO6, POU4F3*, and *SCL26A5* showed significant associations with ARHL for both the single and rare-variant aggregate analyses. Missense variants in *MYO6* [c.737A>G: p.(H246R)] and *POU4F3* [c.694G>C: p.(E232Q)], that showed significant single variant associations, did not drive any rare-variant aggregate associations due to the large number of missense (CADD ≥ 20) variants in *MYO6* (*N* = 431) and *POU4F3* (*N* = 129*)*, which are likely benign. *MYO6* displayed more significant *p*-values when only pLoF variants were analyzed compared to when missense and splice-region (CADD ≥ 20) variants were included. *POU4F3* reached significance exclusively for the pLoF analysis. In contrast, missense variants observed in *CEACAM16* (rs186687142 and rs191552868) and *SLC26A5* (rs141952919) did appear to drive the associations in the rare-variant aggregate association test of pLoF, missense, and splice-region variants (CADD ≥ 20). *CEACAM16* was only exome-wide significant when analyzing this annotation group and *SLC26A5* *p*-values became more significant for every ARHL trait when missense and splice-region variants were included. This agrees well with previous observations in which rare-variant aggregate association tests were only significant for *SCL26A5* and *CEACAM16* when both pLoF and deleterious missense variants were analyzed [7]. This is in line with the fact that *MYO6* [pLI = 0.97; observed/expected (o/e) = 0.38 (0.3–0.48)] and *POU4F3* [pLi = 0.91; o/e = 0.3 (0.17–0.56)] are more intolerant to pLoF variants compared to *SCL26A5* [pLI = 0; o/e = 0.59 (0.46–0.77)] and *CEACAM16* (pLI = 0; o/e = 0.84 (0.63–1.14], as the pLoF-based aggregate association was more significant for *MYO6* and exclusively significant for *POU4F3*.

In this study, additional Mendelian NSHL (i.e., *ACTG1*, *GRHL2, KCNQ4, MYO7A, PLS1*, and *WFS1*) and SHL (i.e. *TNRC6B)* genes that contribute to the risk of developing ARHL were identified through the rare-variant univariate and aggregate analyses. *ACTG1*, *MYO7A*, and *PLS1* genes have been associated with HL closely resembling presbycusis in knockout mouse models [17, 21–24]. *TNRC6B* is a protein-coding gene involved in posttranscriptional gene silencing in association with Argonaut proteins, in which heterozygous mutations are known to cause a developmental syndrome with childhood HL, speech, and language delay [25, 26]. Although, it has not been previously associated with ARHL, common variants in this gene increase susceptibility to tinnitus in UK Biobank patients [27].

We identified several novel ARHL genes using rare-variant aggregate association tests. Among these is *TWF1*, which is widely expressed in the human and mouse inner ear. *TWF1* encodes Twinfilin-1, a highly conserved actin-binding protein that plays a crucial role in cytoskeleton remodeling and is highly expressed in stereocilia [28–30]. In brown-eyed Dalmatian dogs, a variant near *TWF1* was found to be associated with canine congenital sensorineural deafness [31]. Although the mechanism by which Twinfilin-1 underlies ARHL in humans is unknown, it could be due to decreased motility in the hair cells because of altered capping of the actin filaments barbed ends [32]. This process may also be age-dependent; previous studies demonstrated there is overexpression of *Twf1* in cholangiocytes in older mice compared to younger mice [33]. Interestingly, overexpression of Twinfilin-2 (*TWF2* gene) in cochlear inner hair cells (LLC-PK1/CL4) resulted in shorter stereocilia [34].

*PALM3* encodes for paralemmin-3 and has been shown to underlie progressive HL in mice. *PALM3* knock-out mice have severe and progressive early-onset HL by 3 weeks-of-age and are completely deaf by 10 weeks (Personal communication, Vogl and Kilimann 2024). Additionally, the *PALM3* knock-out mouse from the International Mouse Phenotyping Consortium showed decreased or abnormal startle reflex. Expression of PALM3 is restricted to dark cells, involved in the production of endolymph and the inner ear fluid homeostasis, and neurons in humans while in mice its predominantly expressed in inner and outer hair cells [35].

Another newly identified ARHL gene is *FBXO2*, which encodes F-box 2 (FBX2), a member of the F-box protein family. Fbx2 (also known as Fbs1, NFB42, and OCP1) binds high-mannose glycoproteins, targeting them for ubiquitination and degradation via the ubiquitin-proteasome pathway [36, 37]. Initially described as brain-enriched, FBX2 is also highly expressed in the organ of Corti during early otic development in mice [36, 38]. In humans, it is notably expressed in chondrocytes and otic epithelial cells, particularly in the cochlea. Fbxo2^−/−^ mice show normal inner ear morphology and hearing until two months of age, after which they develop age-related cochlear degeneration and HL [39]. Although the mechanism by which FBX2 affects hearing is unknown, previous research suggests that protein quality control is essential for inner ear homeostasis.

*TXNDC17*, also found to be associated with ARHL, encodes thioredoxin domain-containing protein 17, which is a highly conserved and ubiquitously expressed oxidoreductase. TXNDC17 is known to indirectly support glutathione synthesis by efficiently reducing L-cystine [40]. The mechanism by which this gene affects the inner ear causing HL is unclear.

Overall, the highest ORs were observed for the H-aid phenotype, which may be due to hearing aid users having more severe HL than individuals with other self-reported phenotypes. Additionally, some rare-variant aggregate associations (i.e., *MYO7A*, *SIX1*, *TMPRSS3*, *TNRC6B*, and *TXNDC17*) were only detected using SKAT-O, which performs both a random and fixed effects test. These associations were most likely detected through the random effect test, which can be more robust when there are higher proportions of non-causal variants.

Single-variant analysis (MAF > 0.005) identified associations with Mendelian NSHL and SHL genes, some of which have not been reported previously associated with ARHL (i.e., *ABHD12, COA8*, *KANSL1, SERAC1*, and *UBE3B*). In addition, several variants in genes not reported to be involved in Mendelian HL and/or presbycusis were found associated with ARHL (i.e., *ARHGAP27, CRHR1, GOLGB1, GRM3, HCLS1, LRRC37A2, LRRC37A3, MAPT, MMAB, NASP, NSF, PHF23, PLEKHM1, RTRAF, SPPL2C, STH, SYBU* and *VCL*). Interestingly, many of these genes located in region 17q21.31 (i.e., *ARHGAP27, CRHR1, KANSL1, LRRC37A2, MAPT, NSF, PHF23, PLEKHM1, SPPL2C*, and *STH)* have been previously shown to be a complex locus influencing the brain cortical thickness in UK Biobank participants [41]. Further studies are needed to replicate these associations in independent samples and to identify which genes contribute specifically to ARHL risk. This need for replication is especially pertinent for the novel ARHL associations that have not been previously linked to Mendelian HL, as their role in HL in general requires further validation.

Mendelian NSHL and SHL genes play an important role in ARHL. The rare-variant ARHL associations within these genes and other associated genes generally have higher effect sizes than those observed for common variants. Higher frequency variants in Mendelian NSHL and SHL genes also play a role in ARHL, but these variants have lower effect sizes than those observed for rare variants. For certain Mendelian ADNSHL genes associated with ARHL, which have a post-lingual age of onset in Mendelian families, it is possible that the variants may exhibit variable penetrance, leading to some individuals experiencing effects later in life. However, in many cases, variants in Mendelian NSHL and SHL genes that underlie ARHL will not overlap with those that underlie Mendelian forms of HL.

It may be of concern that some of the observed associations are due to accidental inclusions of individuals with early-onset HL even after stringent quality control. Almost all the Mendelian NSHL and SHL genes are rare causes of disease. Additionally, common variants MAF > 0.005 usually do not underlie Mendelian phenotypes. There is one exception in the associations with ARHL that were observed which was with the pathogenic *GJB2* 35delG variant which underlies autosomal recessive prelingual moderate to profound HL across all frequencies and has a MAF in Europeans of ~1%. Although significant associations were observed for this variant with H-diff, there was no significant association with *H-aid* which would be expected for a more severe HL phenotype (Table 3). It is important for future studies to confirm whether this common cause of autosomal recessive NSHL also underlies ARHL etiology. For variants in Mendelian HL genes that are associated with ARHL the age-at-onset of HL may be dependent on the variant and/or genetic background or environmental exposures.

Knowledge on the etiology of ARHL has increased greatly in recent years. However, studies have mainly been limited to Europeans and it is unknown how much overlap there is in the genetic architecture of ARHL between European and other populations. The study of whole-genome sequence data should also provide additional insights through the discovery of ARHL susceptibly variants outside of the coding region as well as better detection of structural variants which are known to play an important role in Mendelian HL. Elucidating the genetic etiology of ARHL should aid in early detection and improved management.

## Web resources

Gene expression analysis resource (gEAR) https://umgear.org/

Genome aggregation database (gnomAD): https://gnomad.broadinstitute.org/

Hereditary hearing loss homepage: https://hereditaryhearingloss.org/

International mouse phenotyping consortium (IMPC): https://www.mousephenotype.org/

Online inheritance in man (OMIM): http://www.omim.org/

UK Biobank: https://www.ukbiobank.ac.uk/

## Supplementary information

Supplemental Figures

Supplemental Table 1

Supplemental Table S2

## Data Availability

Individual-level sequence and phenotype data, from which we derived the traits studied here, are available to approved researchers in the UK Biobank repository. Instructions for access to UK Biobank data are available at https://www.ukbiobank.ac.uk/enable-your-research. Summary statistics are available in the GWAS Catalog [accession numbers are for single variants (GCST90451646-49) and for rare-variant aggregate analyses (GCST90451650-53)]. The code used to perform the analyzes and construct the figures is available at https://github.com/statgenetics/Mendelian-ARHL.
