# Supplementary material for: Mendelian non-syndromic and syndromic hearing loss genes contribute to presbycusis
Source: Eur J Hum Genet. 2025 Mar 7;33(6):758–67. doi: 10.1038/s41431-025-01789-x (PMC12185688; doi:10.1038/s41431-025-01789-x)
Supplement: Supplementary file 1 — Supplemental Figures [file 41431_2025_1789_MOESM1_ESM.docx]

| A. | B. |
| --- | --- |
| 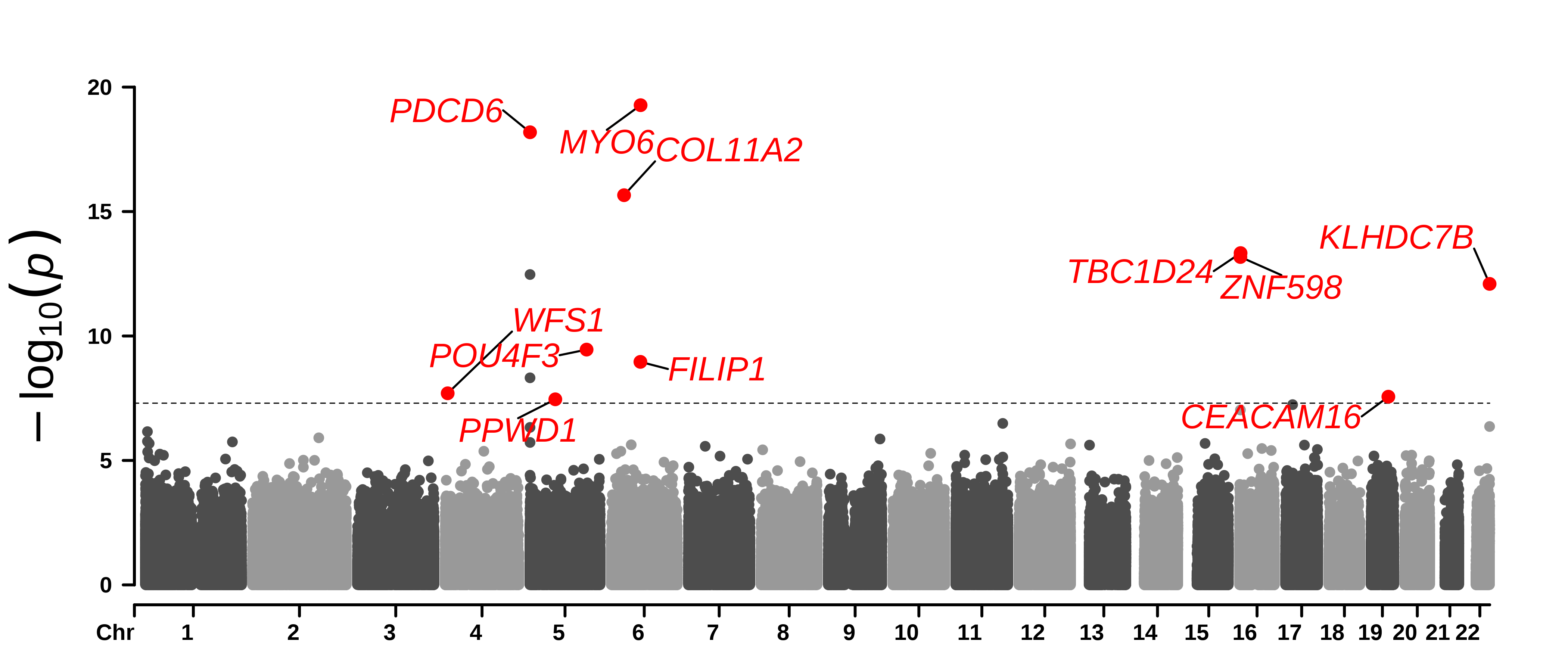 | 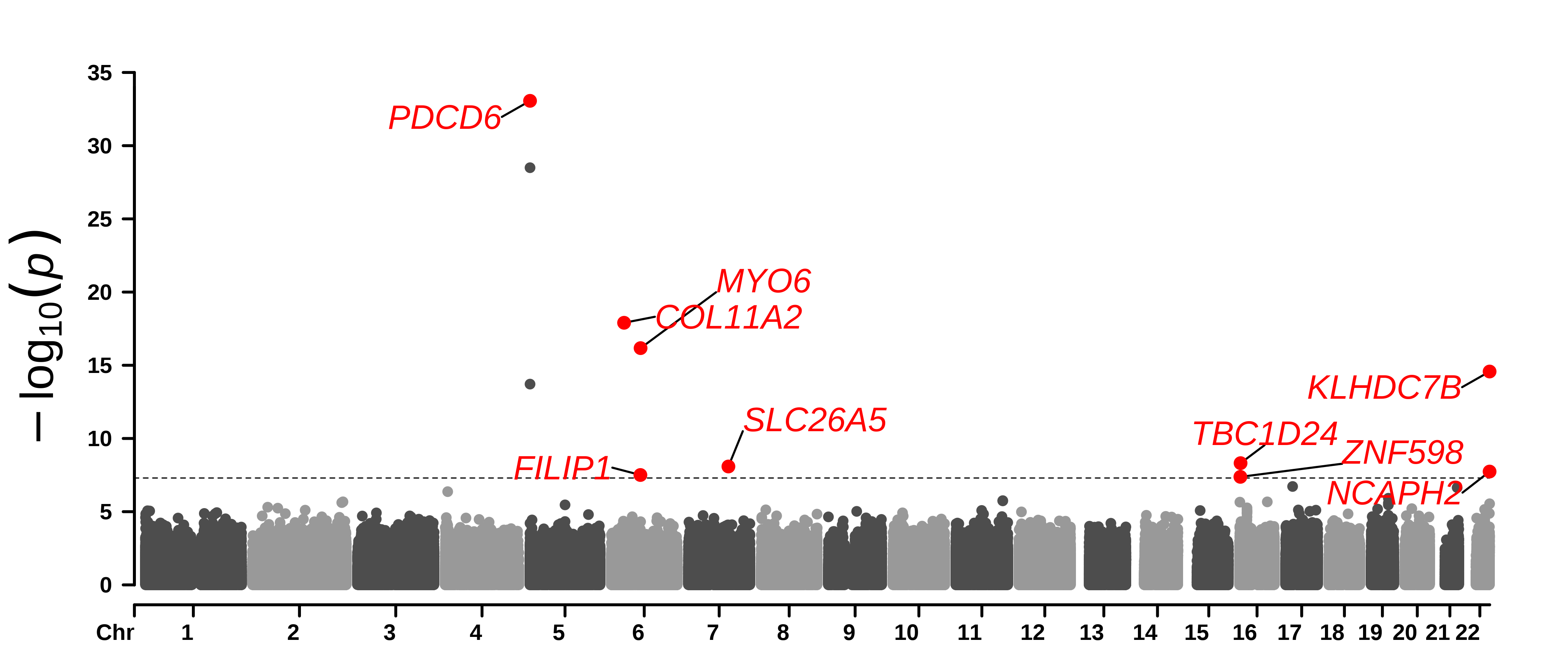 |
| C. | D. |
| 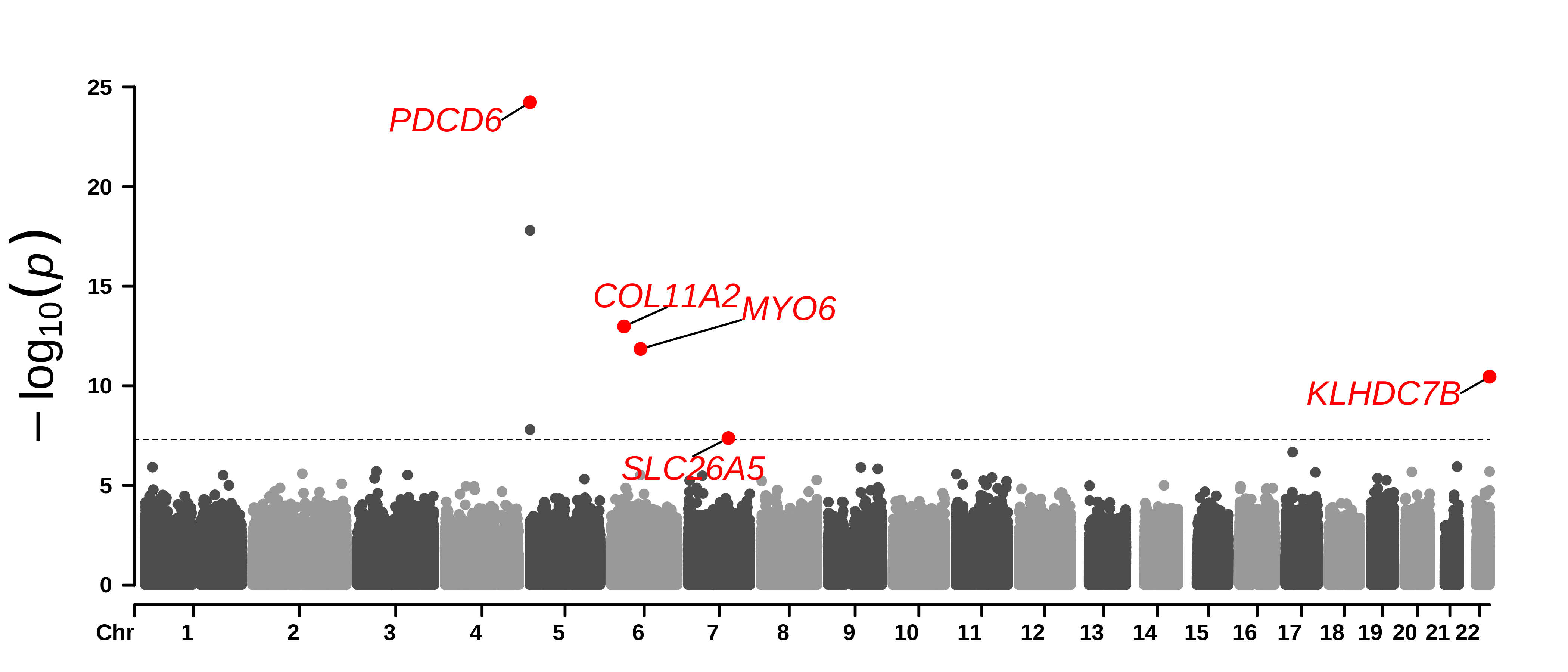 | 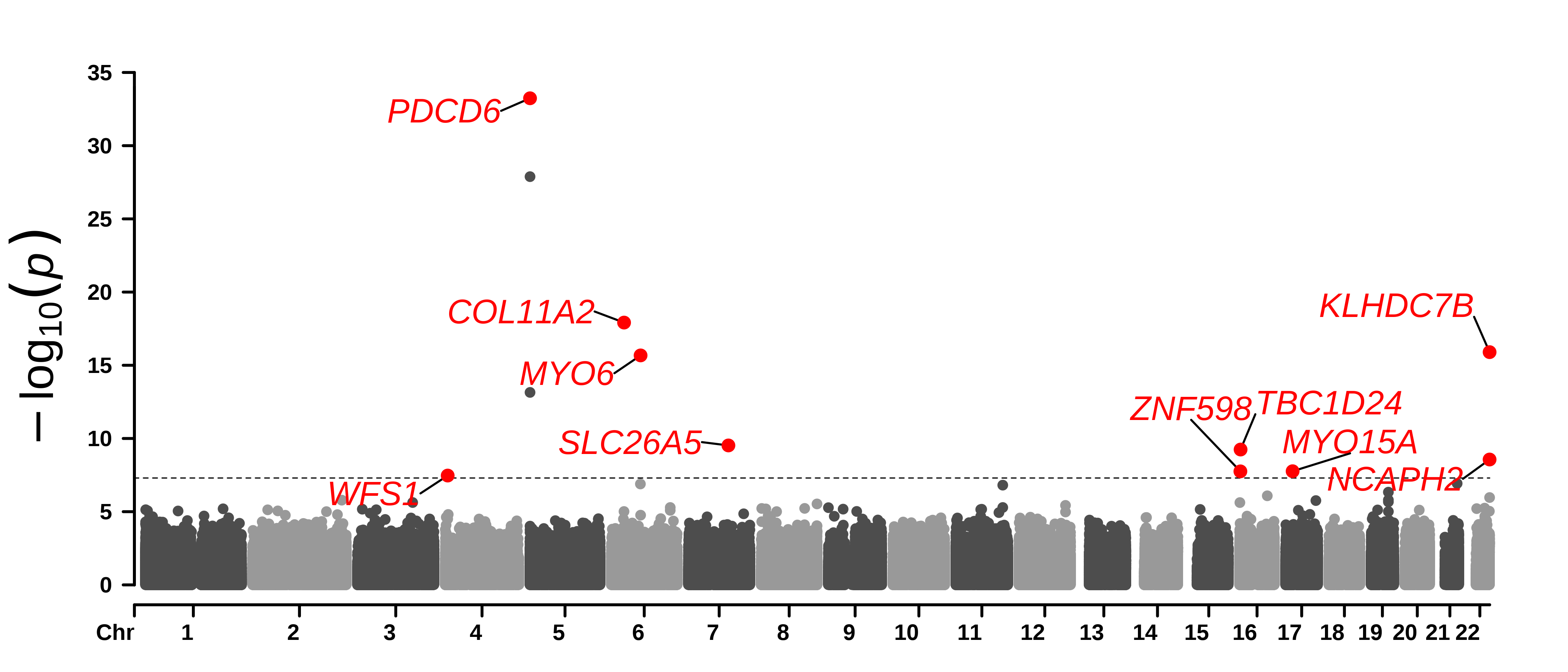 |

**Figure S1. Manhattan plots for single variant analysis of variants with minor allele frequency (MAF)≤ 0.005 for age-related hearing loss.** (A) H-aid, (B) H-diff, (C) H-noise and (D) H-both. The threshold for significance is indicated as a dashed ($p<5\times{10}^{-8}$) line. Genes with variant(s) with MAF≤ 0.005 that reach the genome-wide significance level ($p<5\times{10}^{-8}$) are annotated in red in each Manhattan plot.

| A. | B. |
| --- | --- |
| 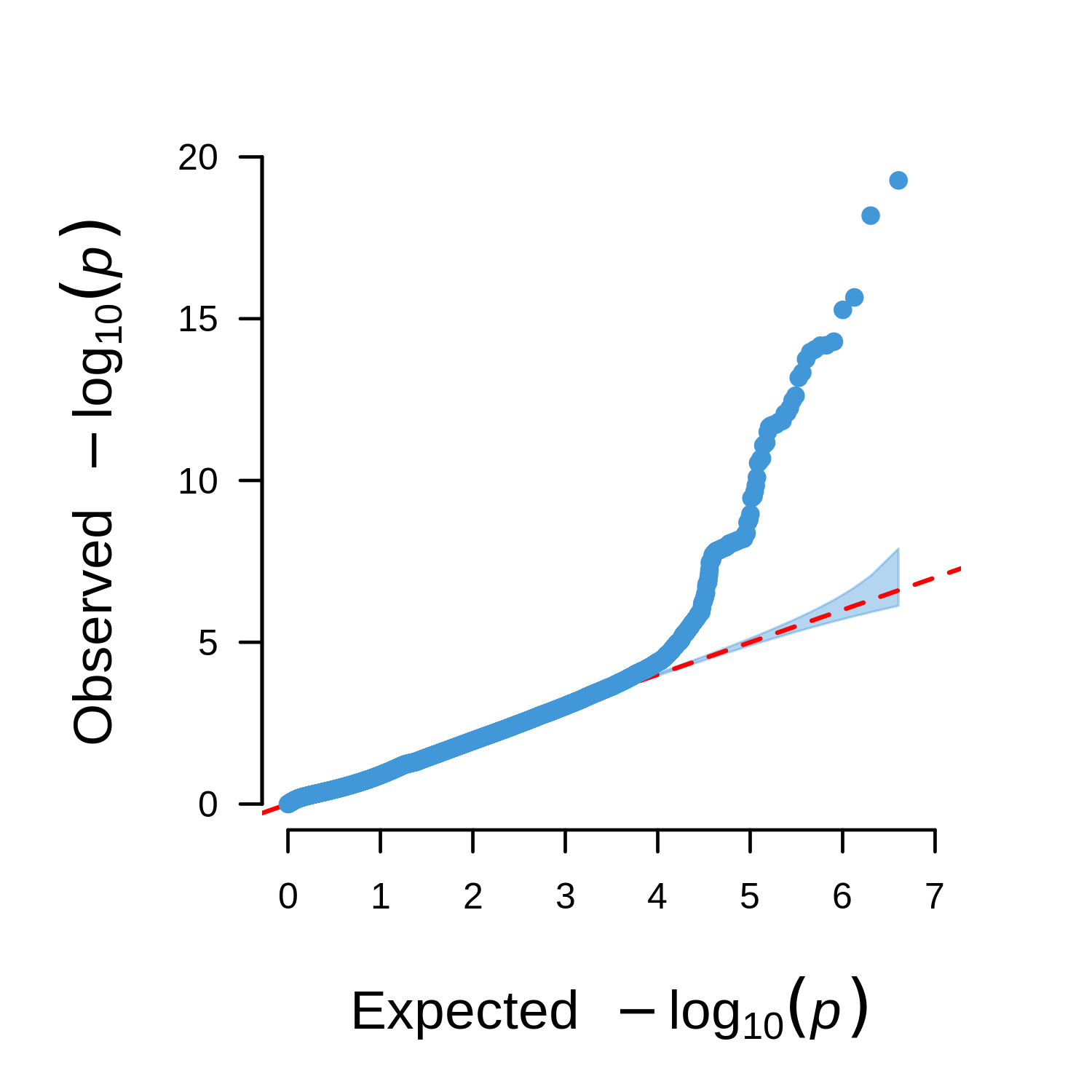 | 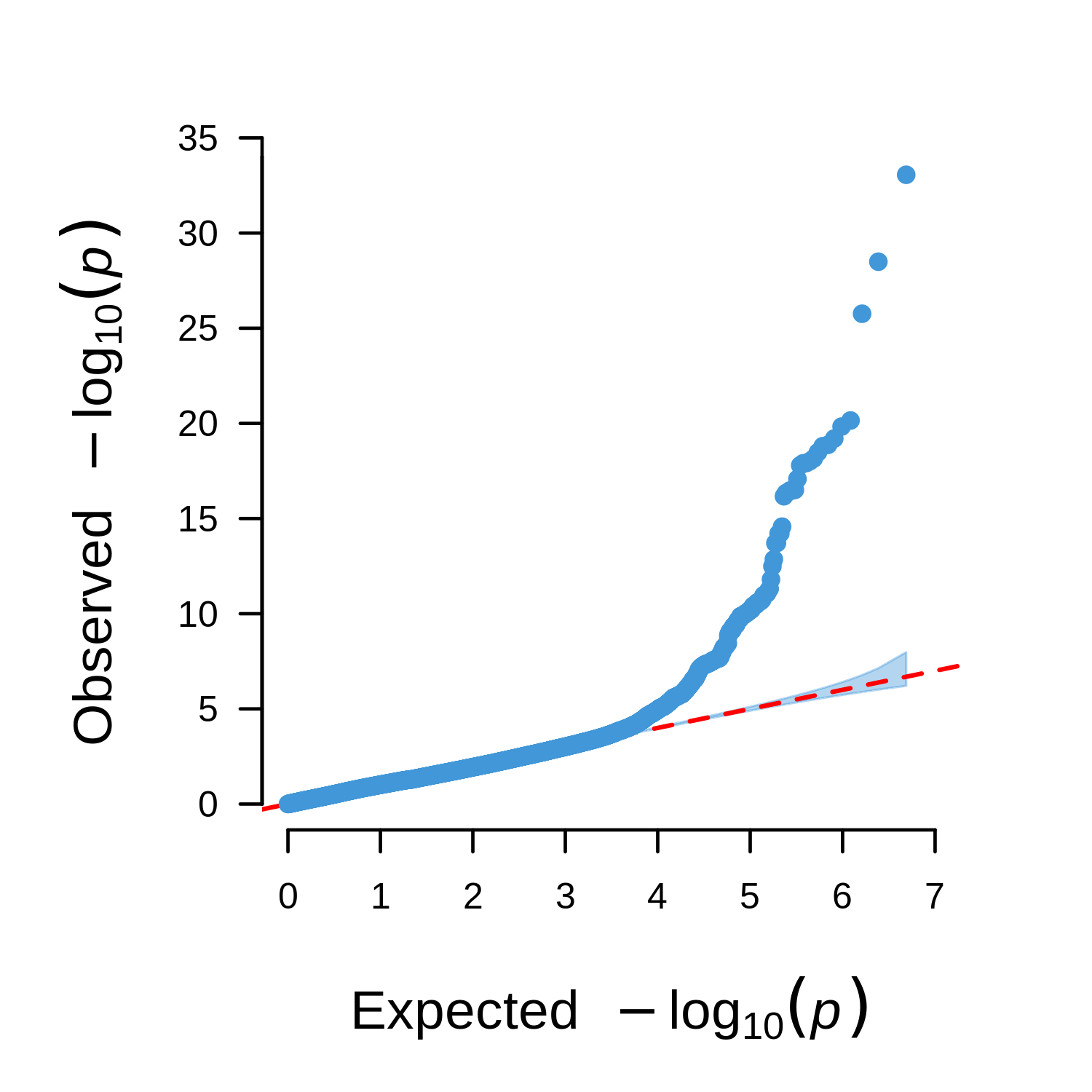 |
| C. | D. |
| 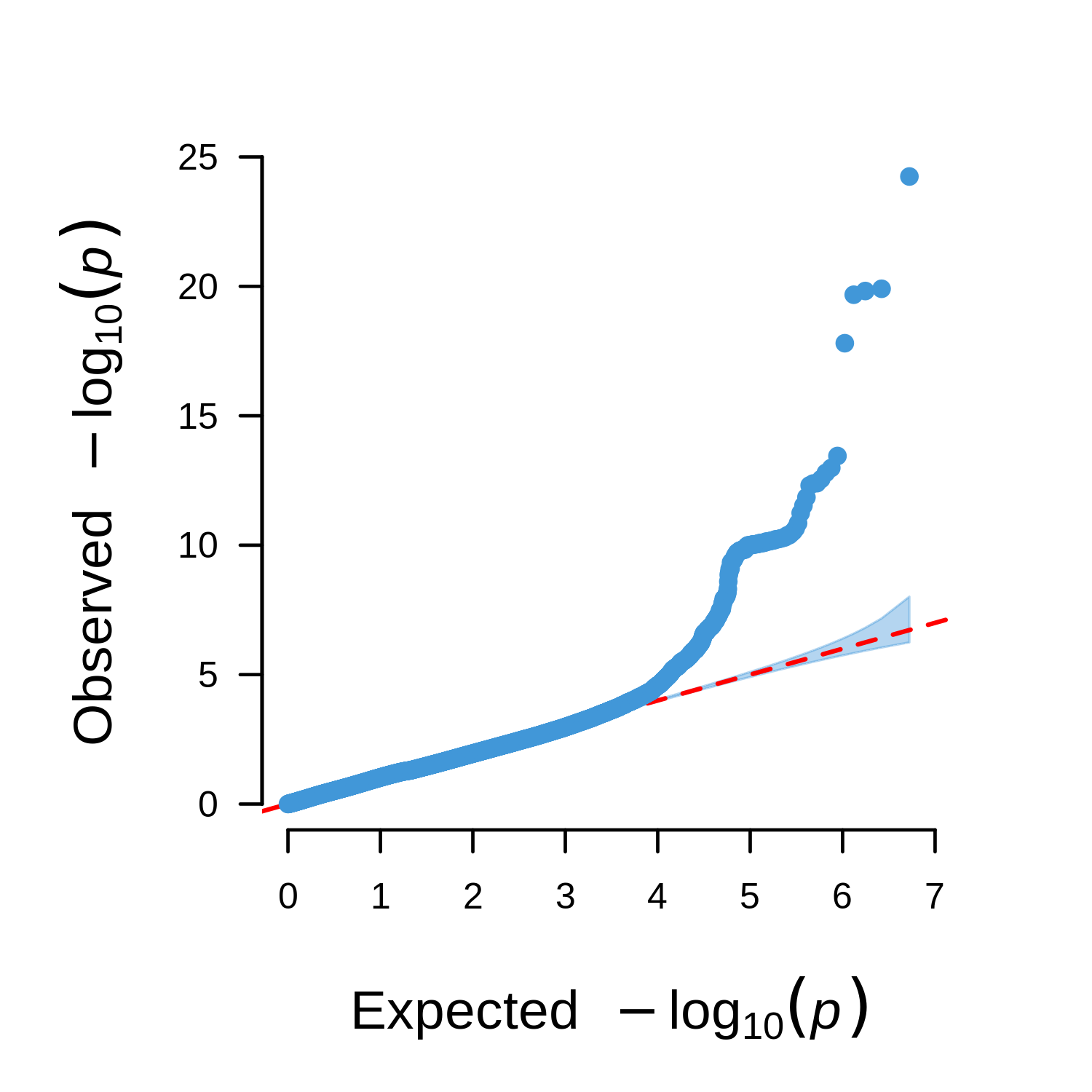 | 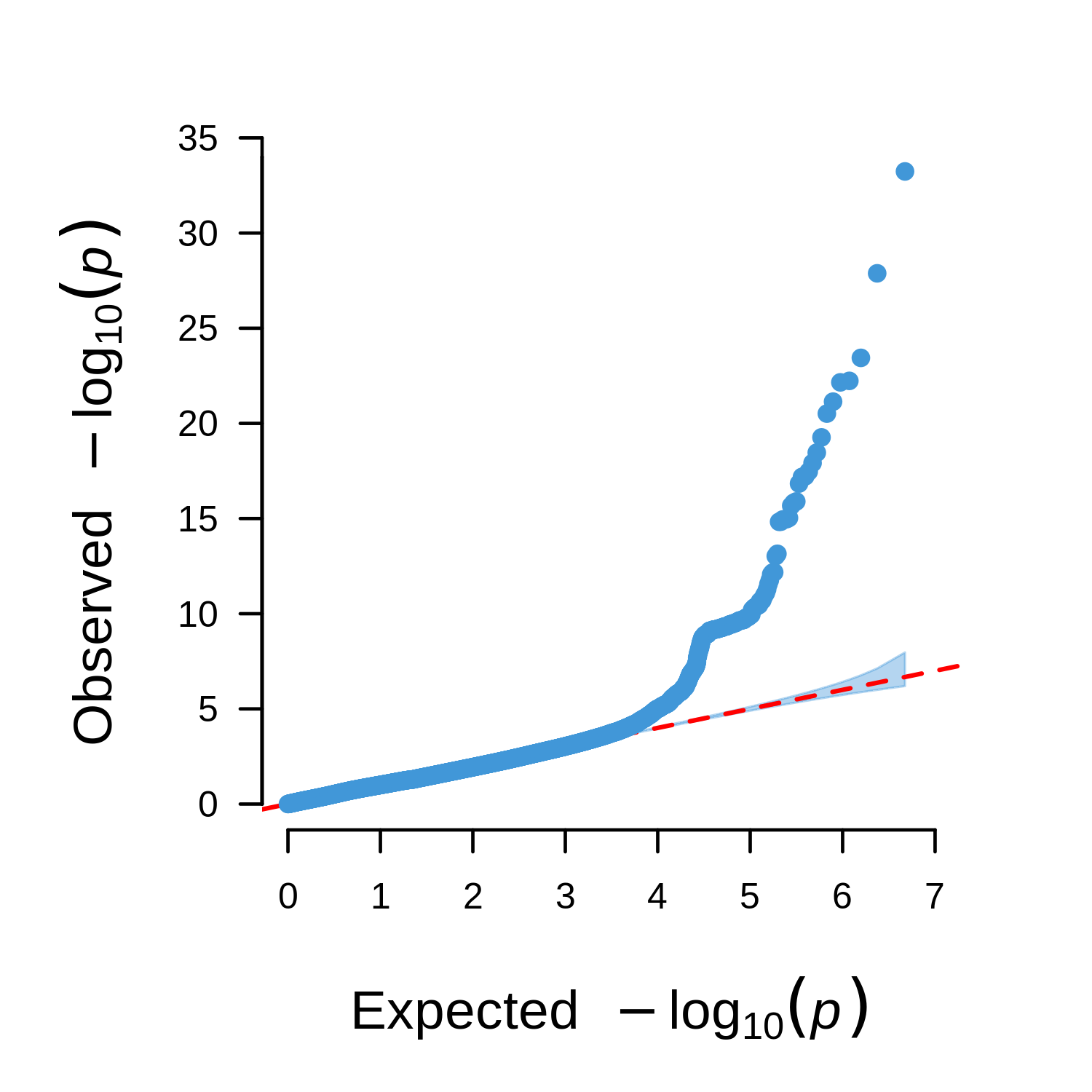 |

**Figure S2. Quantile-Quantile (Q-Q) plots for the single variant association analysis of age-related hearing loss.** (A) H-aid ($\lambda=1.076)$, (B) H-diff ($\lambda=1.048$), (C) H-noise ($\lambda=1.083$) and (D) H-both ($\lambda=1.036$).

| A. | B. |
| --- | --- |
| 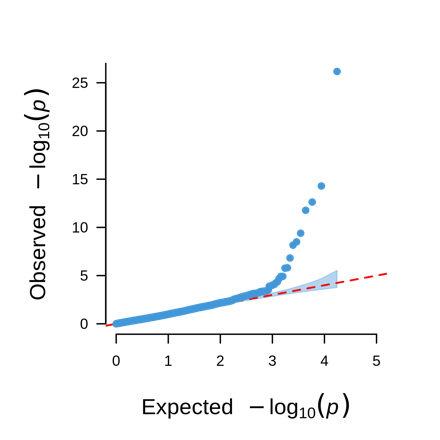 | 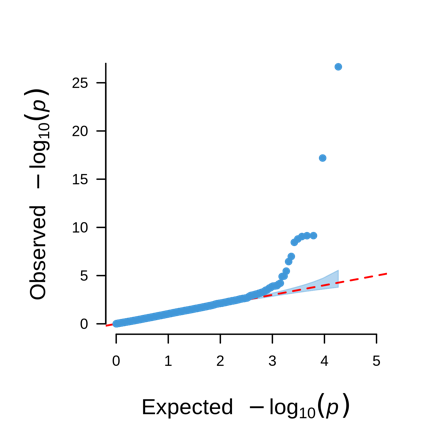 |
| C. | D. |
| 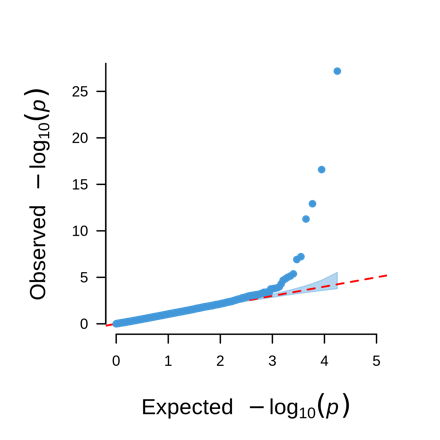 | 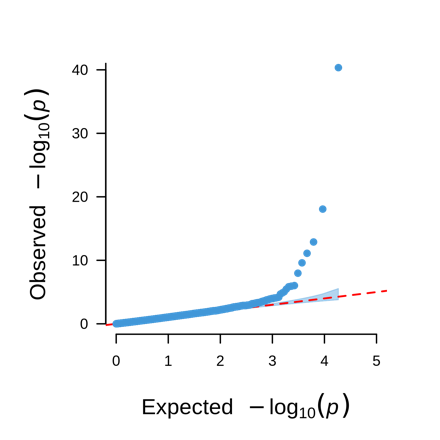 |
| E. | F. |
| 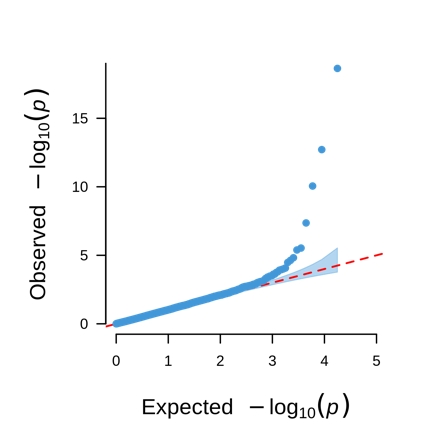 | 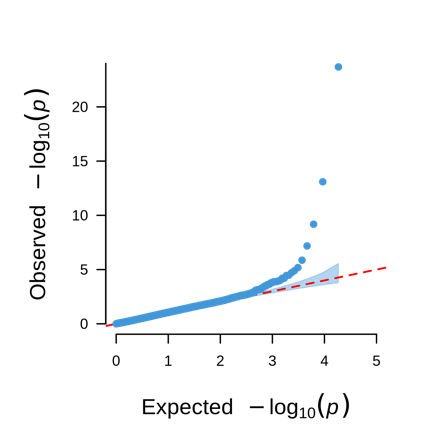 |
| G. | H. |
| 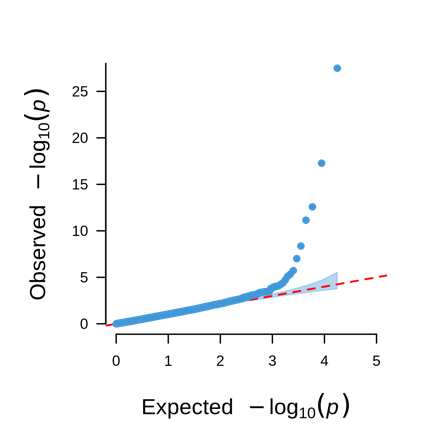 | 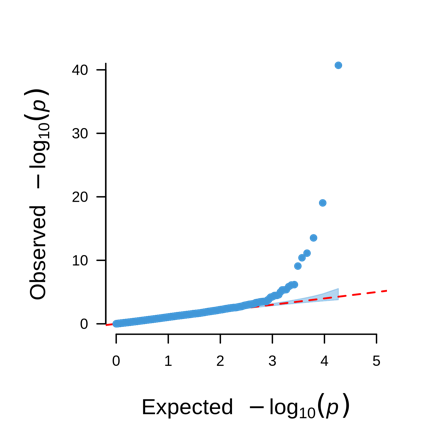 |

**Figure S3. Quantile-Quantile (Q-Q) plots for the SKAT-O rare-variant aggregate association analysis of age-related hearing loss.** H-aid [A: predicted loss of function (pLoF) $\lambda=1.02$ B: pLoF and missense and splice-region (CADD>20) $\lambda=0.95$], H-diff (C: pLoF: $\lambda=0.98$ D: pLoF and missense and splice-region (CADD>20) $\lambda=1.04$), H-noise (E: pLoF $\lambda=0.98$ F: pLoF and missense and splice-region (CADD>20) $\lambda=1.01$) and H-both (G: pLoF $\lambda=0.98$, H: pLoF and missense and splice-region (CADD>20) $\lambda=0.99$).

|  |  |
| --- | --- |
| 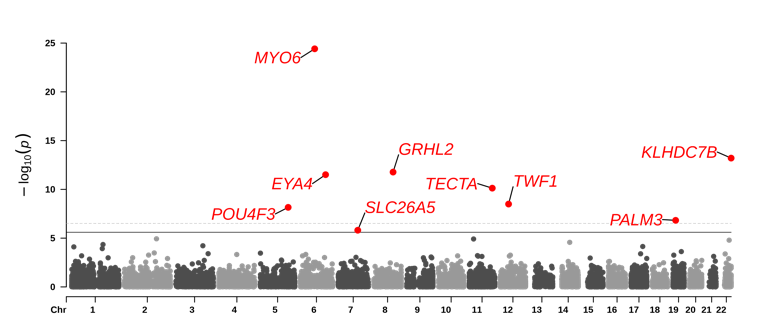 | 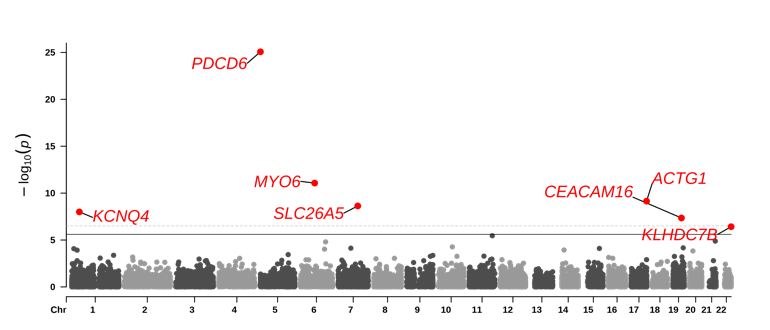 |
|  |  |
| 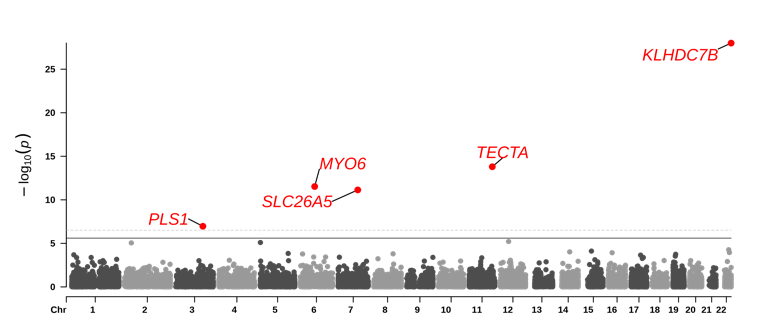 | 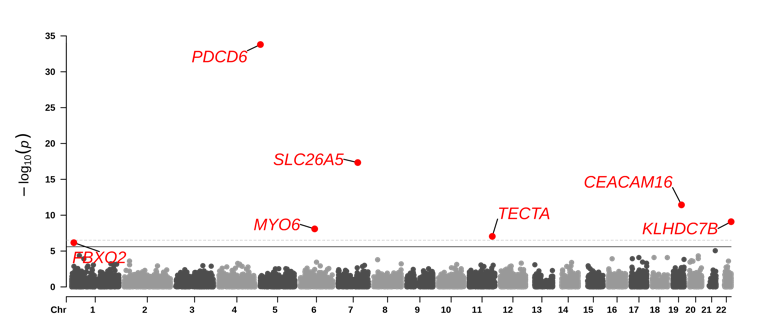 |
|  |  |
| 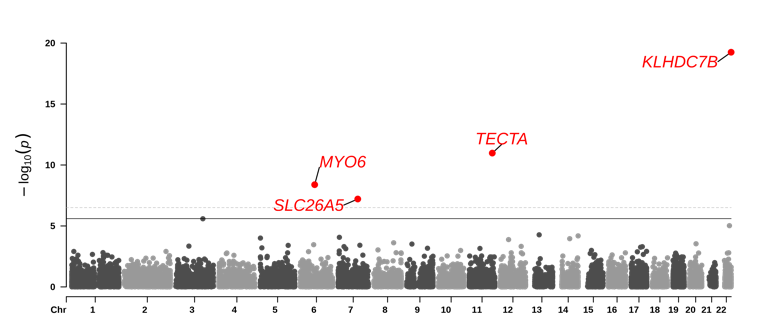 | 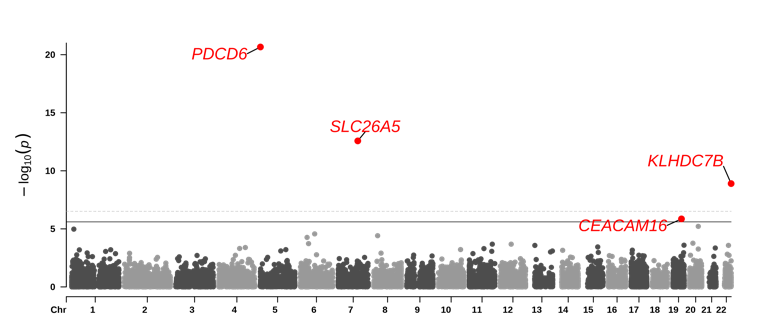 |
|  |  |
| 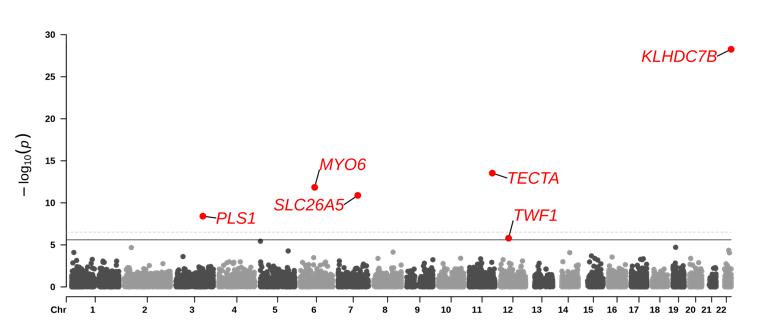 | 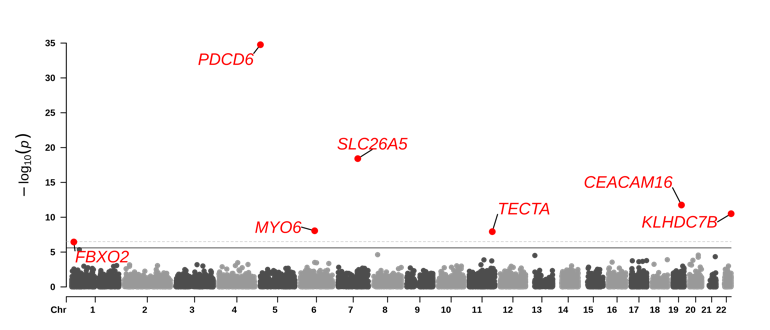 |

**Figure S4.** **Manhattan plots for the burden rare-variant aggregate analysis of age-related hearing loss.** H-aid for (A) the predicted Loss of Function (pLoF) and (B) combined pLoF, missense, and splice-region variants with CADD≥20, H-diff for (C) pLoF and (D) combined pLoF, missense, and splice-region variants with CADD≥20, H-noise for (E) pLoF and (F) combined pLoF, missense, and splice-region variants with CADD≥20, and H-both for the (G) pLoF and (H) combined pLoF, missense, and splice-region variants with CADD≥20. The significance thresholds are shown as follows: a dashed line represents the Bonferroni correction for testing 20,000 genes, four traits, and two variant categories, e.g., pLoF ($p<3.1\times{10}^{-7}$), while a solid line represents the Bonferroni correction for testing 20,000 genes ($p<2.5\times{10}^{-6}$). Genes that reach the exome-wide significance level ($p<2.5\times{10}^{-6}$) are annotated in red in each Manhattan plot.

| A. | B. |
| --- | --- |
| 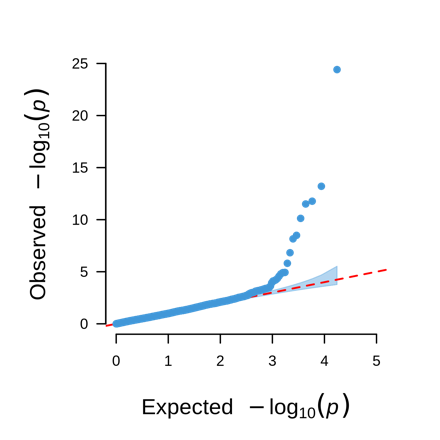 | 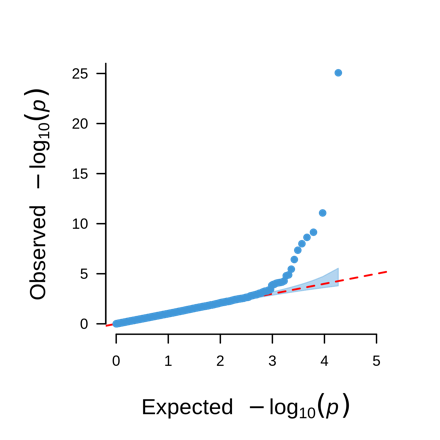 |
| C. | D. |
| 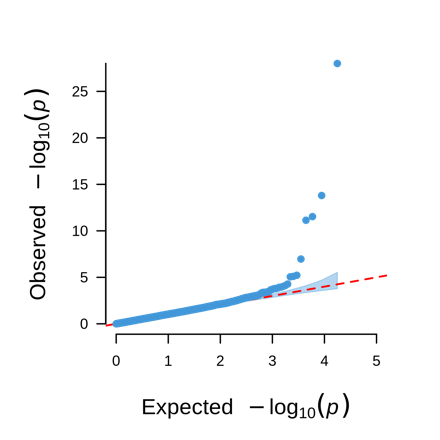 | 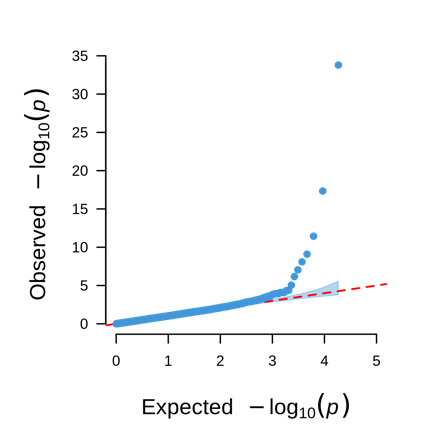 |
| E. | F. |
| 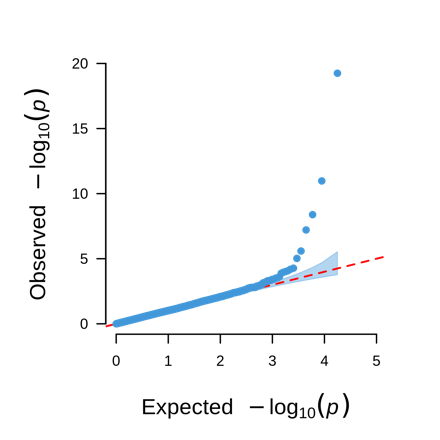 | 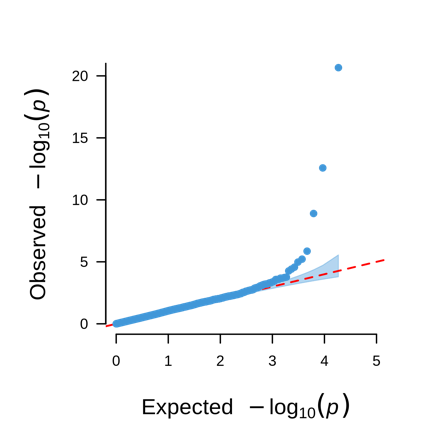 |
| G. | H. |
| 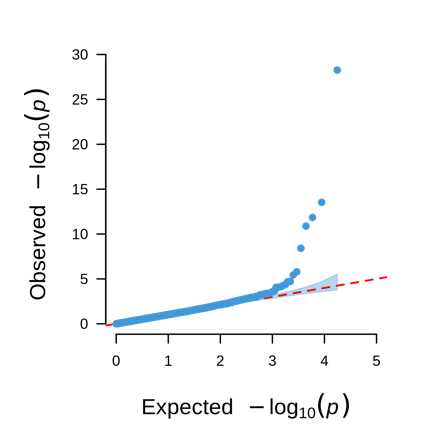 | 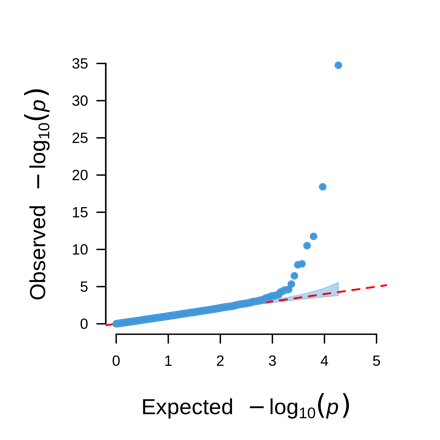 |

**Figure S5. Quantile-Quantile (Q-Q) plots for the burden rare-variant aggregate association analysis of age-related hearing loss.** H-aid [A: predicted loss of function (pLoF) $\lambda=1.08$ B: pLoF and missense and splice-region (CADD>20) $\lambda=1.04$], H-diff (C: pLoF: $\lambda=1.04$ D: pLoF and missense and splice-region (CADD>20) $\lambda=1.02$), H-noise (E: pLoF $\lambda=1.03$ F: pLoF and missense and splice-region (CADD>20) $\lambda=1.03$) and H-both (G: pLoF $\lambda=1.04$, H: pLoF and missense and splice-region (CADD>20) $\lambda=1.04$).


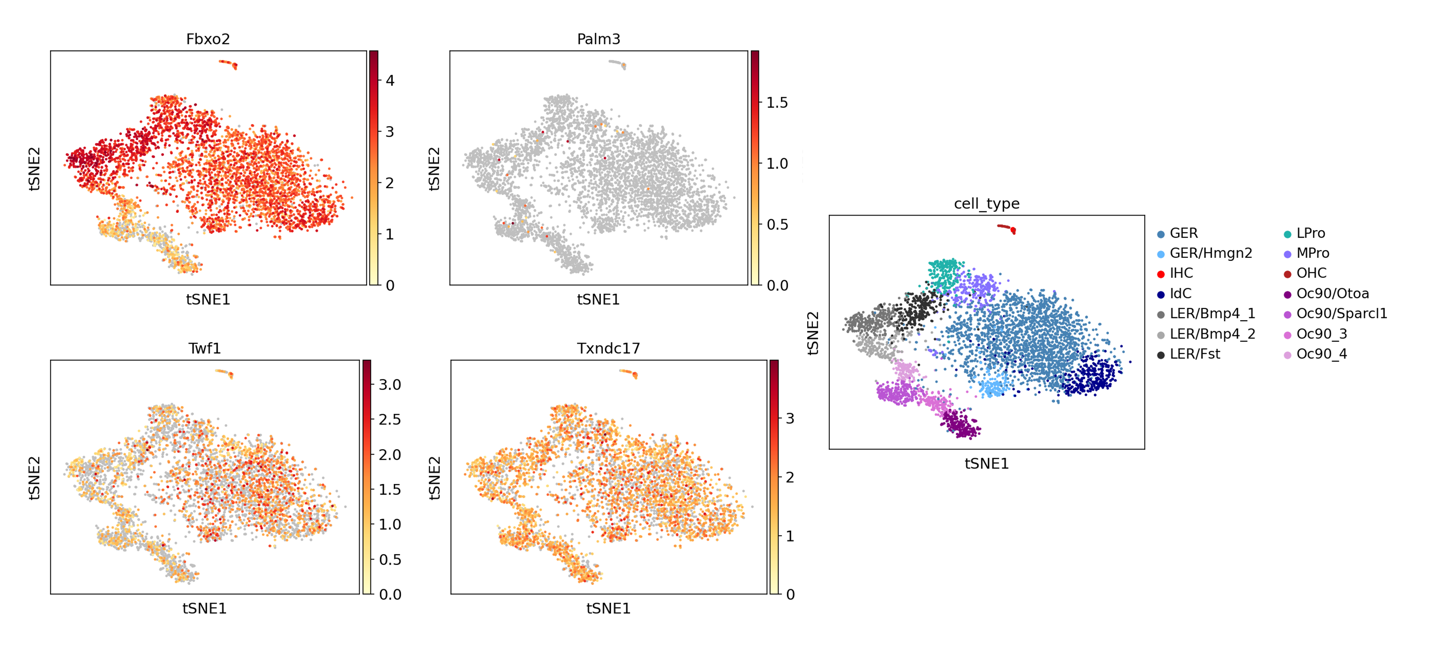


**Figure S6. tSNE plot of the mouse cochlear epithelium at E14 stage illustrating the expression of *Fbxo2, Palm3, Twf1* and *Txndc17***

The dataset includes cells from the cochlear floor epithelium of E14 litters from two wild-type timed-pregnant CD-1 females, with each litter containing 10-12 pups of both sexes. The cell type panel (on the right side) shows the specific locations of each cell type. Tissue codes: GER (Greater Epithelial Ridge), GER/Hmgn2 (Greater Epithelial Ridge expressing Hmgn2), IHC (Inner Hair Cells), IdC (Interdental Cells), LER/Bmp4_1*, LER/Bmp4_2* (Lesser Epithelial Ridge cells expressing Bmp4), LER/Fst (Lesser Epithelial Ridge cells expression Fst), LPro (Lateral Prosensory Cells), MPro (Medial Prosensory Cells), OHC (Outer Hair Cells), Oc90/Otoa # (Cells expressing Oc90 and Otoa), Oc90/Sparcl1 # (Cells expressing Oc90 and Sparcl1), Oc90_3 #* (Cells expressing Oc90), Oc90_4 #* (Cells expressing Oc90) (* Distinct clusters identified by Seurat v2.0, some expressing similar markers like Bmp4 or Oc90, # Oc90+ cells may be early Reissner’s membrane). The scale bar represents gene expression ranging from low-yellow to high-red based on log transformed, normalized, and scaled for sequencing depth expression data.


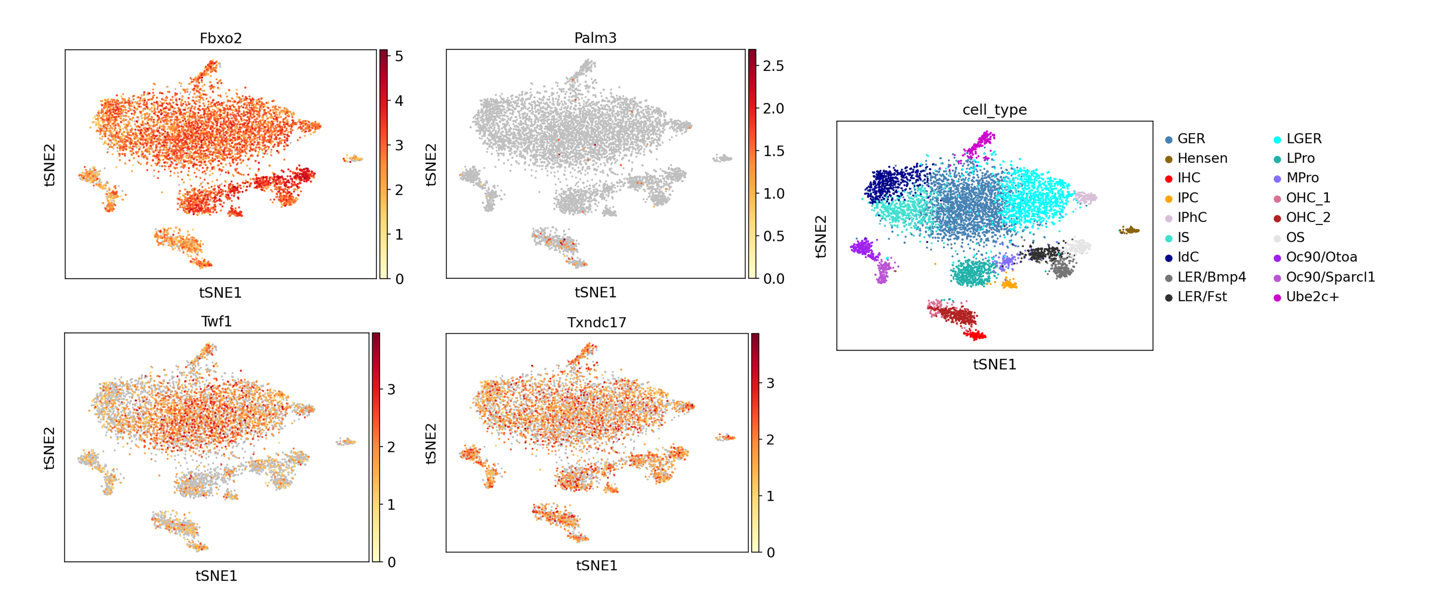


**Figure S7.** **tSNE plots of the mouse cochlear epithelium at E16 stage illustrating the expression of *Fbxo2, Palm3, Twf1* and *Txndc17***

The dataset includes cells from the cochlear floor epithelia duct of E16 stage litters of three wild type timed-pregnant CD-1 females. Each litter had 10-12 pups of both sexes. The cell type panel (on the right side) illustrates the location of each specific cell type. Tissue codes: GER (Greater Epithelial Ridge), Hensen (Hensen Cells), IHC (Inner Hair Cells), IPC (Inner Pillar Cells), IPhC (Inner Phalangeal Cells), IS (Inner Sulcus Cells), IdC (Interdental Cells), LER/Bmp4 (Lesser Epithelial Ridge cells expressing Bmp4), LER/Fst (Lesser Epithelial Ridge cells expression Fst), LGER (Lateral Greater Epithelial Ridge Cells), LPro (Lateral Prosensory Cells), MPro (Medial Prosensory Cells), OHC_1 (More mature developing outer hair cells), OHC_2 (Less mature developing outer hair cells), OS (Outer Sulcus Cells), Oc90/Otoa (Cells expressing Oc90 and Otoa), (Oc90/Sparcl1) Cells expressing Oc90 and Sparcl1, Ube2c+ (Unannotated cells expressing Ube2c+). The scale bar represents gene expression ranging from low-yellow to high-red based on log transformed, normalized, and scaled for sequencing depth expression data.


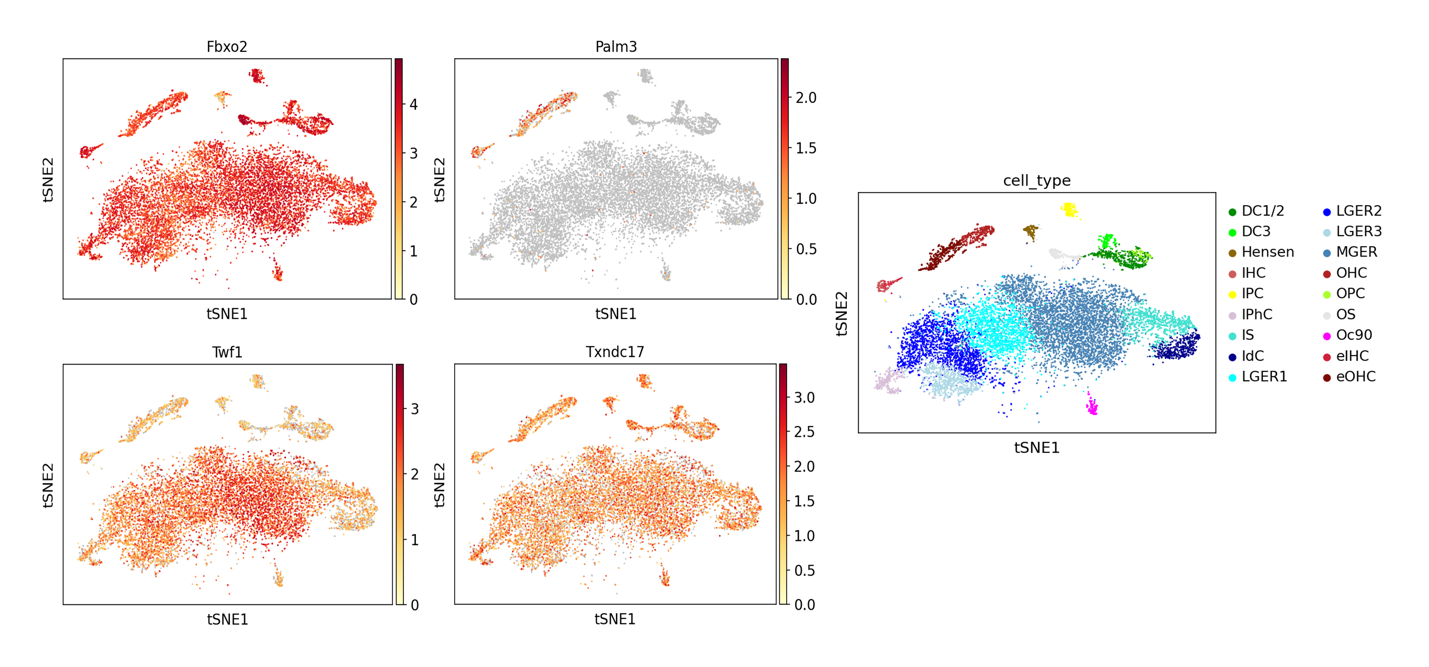


**Figure S8.** **tSNE plots of the mouse cochlear epithelium at P1 stage illustrating the expression of *Fbxo2, Palm3, Twf1* and *Txndc17***

The dataset includes cells obtained from the cochlear floor epithelia duct of ~20-32 P1 CD-1 pups of both sexes. The cell type panel (on the right side) illustrates the location and cell type. Tissue codes: Developing supporting cells- DC1/2 (Deiters’ cells rows 1 and 2), DC3 (Deiters’ cells row 3), Hensen (Hensen’s cells), IPC (inner pillar cells), IPhC (inner phalangeal cells/border cells), IS (inner sulcus cells), IdC (interdental cells), OPC (outer pillar cells), OS (Outer sulcus cells), Oc90 (OC90^+^ cells). Developing greater epithelial ridge cells- LGER1, LGER2, LGER3, MGER. Developing sensory cells- IHC (Inner hair cells), OHC (Outer hair cells), eIHC, eOHC. The scale bar represents gene expression ranging from low-yellow to high-red based on log transformed, normalized, and scaled for sequencing depth expression data.


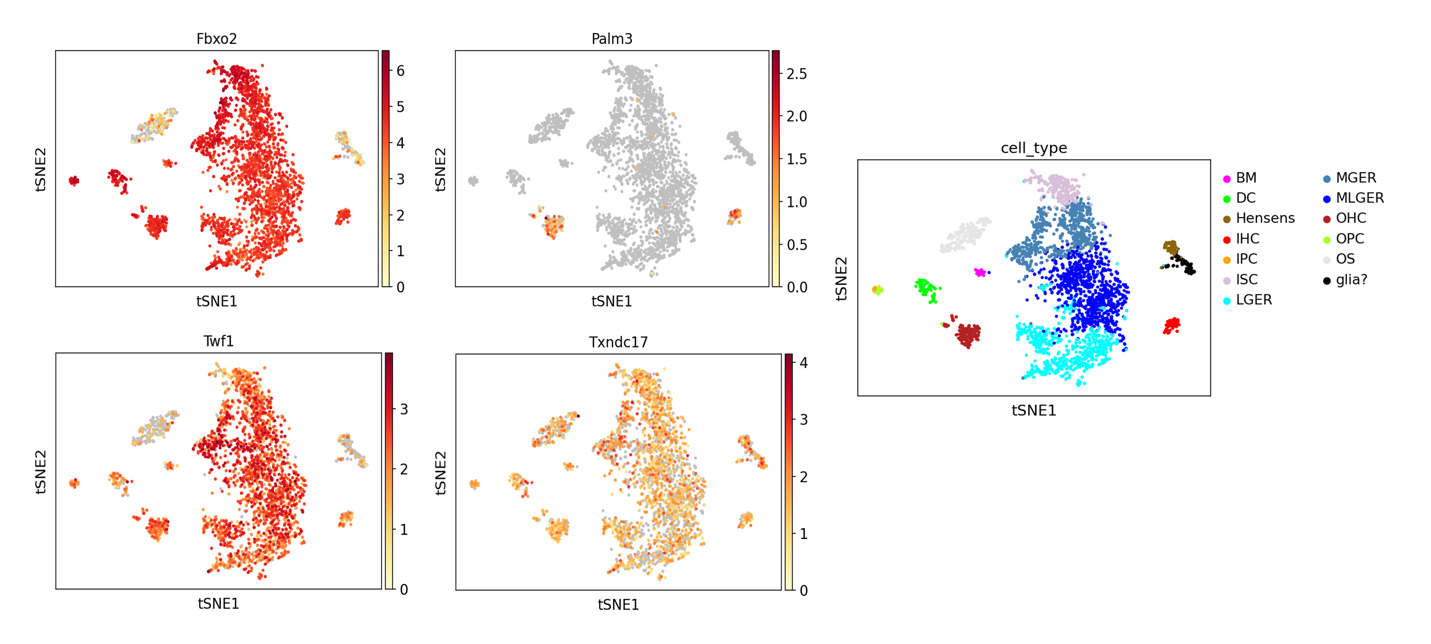


**Figure S9.** **tSNE plots of the mouse cochlear epithelium at P7 stage illustrating the expression of *Fbxo2, Palm3, Twf1* and *Txndc17***

The dataset includes cells obtained from the cochlear floor epithelia duct of ~15-24 P7 CD-1 pups of both sexes. The cell type panel (on the right side) illustrates the location and cell. Tissue codes: BM (Basilar Membrane cells), DC (Deiters’ cells), Hensen (Hensen’s cells), IHC (Inner Hair Cells), IPC (Inner Pillar Cells), ISC (Inner Sulcus Cells), LGER (Lateral Greater Epithelial Ridge Cells), MGER (Medial Greater Epithelial Ridge Cells), MLGER (Medial Lateral Greater Epithelial Ridge Cells), OHC (Outer hair cells), OPC (outer pillar cells), OS (Outer sulcus cells), Glia (Glial cells). The scale bar represents gene expression ranging from low-yellow to high-red based on log transformed, normalized, and scaled for sequencing depth expression data.
