# Supplementary material for: Mendelian non-syndromic and syndromic hearing loss genes contribute to presbycusis
Source: Eur J Hum Genet. 2025 Mar 7;33(6):758–67. doi: 10.1038/s41431-025-01789-x (PMC12185688; doi:10.1038/s41431-025-01789-x)
Supplement: Supplementary file 2 — Supplemental Table 1 [file 41431_2025_1789_MOESM2_ESM.docx]

| **Table S1: Burden rare-variant association results with age-related hearing loss phenotypes** | | | | | | | | | | |
| --- | --- | --- | --- | --- | --- | --- | --- | --- | --- | --- |
| **pLoF variants** | | | | | | | | | | |
| **Mendelian HL genes associated with ARHL** | | | | | | | | | | |
| **Chr** | **Gene** | **H-aid** | | **H-diff** | | **H-noise** | | **H-both** | | **HL Phenotypes^1,2^** |
|  |  | **β(SE)** | **P-value** | **β (SE)** | **P-value** | **β (SE)** | **P-value** | **β (SE)** | **P-value** |  |
| 3 | *PLS1* | 0.98(0.23) | 6.0×10^-5^ | 0.63(0.12) | **1.1×10^-7***^** | 0.51(0.11) | 2.6×10^-6^ | 0.71(0.12) | **3.9×10^-9***^** | DFNA76 |
| 5 | *POU4F3* | 6.19(1.71) | **7.0×10^-9***^** | 3.65(1.59) | 1.4×10^-4^ | 3.33(1.56) | 3.9×10^-4^ | 3.85(1.60) | 5.3×10^-5^ | DFNA15, ARHL^3^ |
| 6 | *MYO6* | 2.44(0.21) | **3.9×10^-25***^** | 1.07(0.15) | **3.0×10^-12***^** | 0.85(0.15) | **4.1×10^-9***^** | 1.12(0.16) | **1.4×10^-12***^** | DFNA22; DFNB37, ARHL^3,4^ |
| 6 | *EYA4* | 2.36(0.31) | **3.1×10^-12***^** | 0.78(0.22) | 3.7×10^-4^ | 0.54(0.21) | 9.8×10^-3^ | 0.76(0.23) | 1.1×10^-3^ | DFNA10, ARHL^3,4^ |
| 7 | *SLC26A5* | 1.79(0.34) | **1.6×10^-6*^** | 1.33(0.20) | **7.3×10^-12***^** | 1.00(0.19) | **6.2×10^-8***^** | 1.36(0.20) | **1.3×10^-11***^** | DFNB61, ARHL^4^ |
| 8 | *GRHL2* | 3.92(0.53) | **1.7×10^-12***^** | 1.58(0.44) | 1.6×10^-4^ | 1.11(0.44) | 7.3×10^-3^ | 1.70(0.45) | 7.3×10^-5^ | DFNA28 |
| 11 | *TECTA* | 1.14(0.16) | **7.5×10^-11***^** | 0.68(0.09) | **1.6×10^-14***^** | 0.55(0.08) | **1.1×10^-11***^** | 0.70(0.09) | **2.9×10^-14***^** | DFNA8/12; DFNB21, ARHL^3,4^ |
| 14 | *SIX1* | 1.89(0.40) | 2.7×10^-5^ | 1.16(0.31) | **9.5×10^-5^** | 1.06(0.29) | 1.1×10^-4^ | 1.20(0.31) | **8.4×10^-5^** | DFNA23; BORS^5^, ARHL^3^ |
| 22 | *TNRC6B* | 1.81(0.40) | **1.6×10^-5^** | 0.60(0.25) | 1.7×10^-2^ | 0.41(0.24) | 8.7×10^-2^ | 0.71(0.25) | 5.9×10^-3^ | Developmental delay^6^ |
| **Novel gene ARHL associations** | | | | | | | | | | |
| 12 | *TWF1* | 2.62(0.42) | **3.2×10^-9***^** | 1.29(0.29) | 6.0×10^-6^ | 1.04(0.28) | 1.3×10^-4^ | 1.39(0.30) | **1.6×10^-6**^** | HL in Dalmatian dogs^7^ |
| 19 | *PALM3* | 2.41(0.42) | **1.5×10^-7***^** | 1.00(0.27) | 1.8×10^-4^ | 0.78(0.25) | 1.7×10^-3^ | 1.15(0.27) | 2.0×10^-5^ | Mouse HL gene mice^8^ |
| **Genes previously associations with ARHL** | | | | | | | | | | |
| 22 | *KLHDC7B^3,4^* | 1.04(0.13) | **6.2×10^-14***^** | 0.74(0.07) | **1.0×10^-28 ***^** | 0.56(0.06) | **5.6×10^-20***^** | 0.78(0.07) | **5.5×10^-29***^** |  |
| **pLoF, missense and splice-region variants with CADD≥20** | | | | | | | | | | |
| **Mendelian HL genes associated with ARHL** | | | | | | | | | | |
| 1 | *KCNQ4* | 0.55(0.09) | **1.0×10^-8***^** | 0.19(0.05) | 4.3×10^-5^ | 0.14(0.04) | 6.5×10^-4^ | 0.22(0.05) | **4.6×10^-6^** | DFNA2A |
| 6 | *MYO6* | 0.45(0.06) | **8.5×10^-12***^** | 0.18(0.03) | **8.1×10^-9***^** | 0.12(0.03) | **2.7×10^-5^** | 0.19(0.03) | **8.7×10^-9***^** | DFNA22, DFNB37 |
| 7 | *SLC26A5* | 0.37(0.06) | **2.3×10^-9***^** | 0.25(0.03) | **4.6×10^-18***^** | 0.19(0.03) | **2.7×10^-13***^** | 0.27(0.03) | **3.9×10^-19***^** | DFNB61 |
| 11 | *MYO7A* | 0.12(0.03) | 5.2×10^-4^ | 0.05(0.02) | 1.4×10^-3^ | 0.05(0.01) | 5.0×10^-4^ | 0.06(0.02) | **1.3×10^-4^** | DFNA11, DFNB2, Usher syndrome |
| 11 | *TECTA* | 0.20(0.04) | **3.5×10^-6^** | 0.11(0.02) | **9.0×10^-8***^** | 0.07(0.02) | 2.1×10^-4^ | 0.12(0.02) | **1.2×10^-8***^** | DFNA8/12, DFNB21 |
| 17 | *ACTG1* | 1.63(0.24) | **7.2×10^-10***^** | 0.54(0.15) | 5.0×10^-4^ | 0.33(0.14) | 2.1×10^-2^ | 0.60(0.16) | 1.7×10^-4^ | DFNA20/26 |
| 19 | *CEACAM16* | 0.46(0.08) | **4.5×10^-8***^** | 0.27(0.04) | **3.6×10^-12***^** | 0.17(0.04) | **1.4×10^-6**^** | 0.29(0.04) | **1.8×10^-12***^** | DFNA4B; DFNB113 |
| 21 | *TMPRSS3* | 0.25(0.10) | **1.3×10^-5^** | 0.12(0.03) | **8.8×10^-6^** | 0.08(0.02) | 4.5×10^-4^ | 0.11(0.03) | 4.6×10^-5^ | DFNB8/10 |
| **Novel gene-based association** | | | | | | | | | | |
| 1 | *FBXO2* | 0.54(0.13) | 8.4×10^-5^ | 0.32(0.06) | **6.8×10^-7**^** | 0.26(0.06) | 1.1×10^-5^ | 0.35(0.07) | **3.6×10^-7**^** | ARHL in mice^9^ |
| 17 | *TXNDC17* | 0.45(0.29) | 1.2×10^-1^ | 0.43(0.11) | **1.2×10^-4^** | 0.34(0.10) | 6.8×10^-4^ | 0.44(0.12) | 1.8×10^-4^ |  |
| **Genes previously reported to be associated with ARHL** | | | | | | | | | | |
| 5 | *PDCD6^4^* | 1.01(0.09) | **8.5×10^-26***^** | 0.59(0.05) | **1.6×10^-34***^** | 0.42(0.04) | **2.2×10^-21***^** | 0.63(0.05) | **1.7×10^-35***^** |  |
| 22 | *KLHDC7B^3,4^* | 0.35(0.07) | **3.8×10^-7**^** | 0.20(0.03) | **8.1×10^-10***^** | 0.17(0.03) | **1.3×10^-9***^** | 0.22(0.03) | **3.1×10^-11***^** |  |
| These results are for the same genes which are shown in Table 2, but for the burden test for which a β value can be obtained. *Significant after Bonferroni correction for 20,000 genes ($p<2.5\times{10}^{-6})$, **Significant after Bonferroni correction for testing 20,000 genes and testing two categories of variants, e.g., ($p<1.25\times{10}^{-6}$), ***Significant after Bonferroni correction for testing 20,000 genes, four traits, and two variant categories, e.g., pLoF ($p<3.1\times{10}^{-7}$), ^1^DFNA: Autosomal dominant non-syndromic hearing loss locus, ^2^DFNB: Autosomal recessive non-syndromic hearing loss locus, ^3^Praveen et. al 2022 (PMID: 35661827), ^4^Cornejo-Sanchez et al. 2023 (PMID: 36788145), ^5^BORS: Branchio-oto-renal syndrome, ^6^with speech, behavioral abnormalities and hearing loss, ^7^Candidate gene for congenital sensorineural deafness in Dalmatian dogs (PMID: 24324618), ^8^Personal communication Vogl and Kilimann – hearing loss gene in mice. ^9^Deletion of *FBXO2* in mice leads to age-related hearing loss beginning by two months of age (PMID: 17494702). Chr: chromosome, pLoF: predicted loss of function, P-value: p-value for burden test, H-aid: hearing aid, H-diff: hearing difficulty, H-noise: hearing difficulty with background noise, H-both: hearing difficulty and hearing difficulty with background noise. P-values shown in bold are also significant in SKAT-O analyses (see Table 2). | | | | | | | | | | |
